# Cdk8 attenuates lipogenesis by inhibiting SREBP-dependent transcription in *Drosophila*

**DOI:** 10.1242/dmm.049650

**Published:** 2022-11-14

**Authors:** Xiao Li, Meng Zhang, Mengmeng Liu, Tzu-Hao Liu, Rajitha-Udakara-Sampath Hemba-Waduge, Jun-Yuan Ji

**Affiliations:** ^1^ Department of Molecular and Cellular Medicine, College of Medicine, Texas A&M University Health Science Center, College Station, TX 77843, USA; ^2^ Department of Biochemistry and Molecular Biology, Tulane University School of Medicine, Louisiana Cancer Research Center, 1700 Tulane Avenue, New Orleans, LA 70112, USA

**Keywords:** Cdk8, SREBP, Lipogenesis, Mediator complex, *Drosophila*

## Abstract

Fine-tuning of lipogenic gene expression is important for the maintenance of long-term homeostasis of intracellular lipids. The SREBP family of transcription factors are master regulators that control the transcription of lipogenic and cholesterogenic genes, but the mechanisms modulating SREBP-dependent transcription are still not fully understood. We previously reported that CDK8, a subunit of the transcription co-factor Mediator complex, phosphorylates SREBP at a conserved threonine residue. Here, using *Drosophila* as a model system, we observed that the phosphodeficient SREBP proteins (SREBP-Thr390Ala) were more stable and more potent in stimulating the expression of lipogenic genes and promoting lipogenesis *in vivo* than wild-type SREBP. In addition, starvation blocked the effects of wild-type SREBP-induced lipogenic gene transcription, whereas phosphodeficient SREBP was resistant to this effect. Furthermore, our biochemical analyses identified six highly conserved amino acid residues in the N-terminus disordered region of SREBP that are required for its interactions with both Cdk8 and the MED15 subunit of the small Mediator complex. These results support that the concerted actions of Cdk8 and MED15 are essential for the tight regulation of SREBP-dependent transcription.

This article has an associated First Person interview with the first author of the paper.

## Teaser

**Summary:** The concerted actions of Cdk8 and MED15 play critical roles in tight regulation of SREBP-dependent transcription in the nucleus and *de novo* lipogenesis in *Drosophila*.

## INTRODUCTION

Cellular lipids, such as fatty acids, triglycerides, phospholipids and sterols derived from isoprenoids, play diverse and critical roles during the normal development of multicellular organisms. For example, triglycerides serve as the major energy storage molecules; phospholipids and sterols form membrane structures that are essential for compartmentation of eukaryotic cells; and phosphatidylinositols, cholesterol and its derivatives can also function as signaling molecules or hormones in multicellular organisms (Fahy et al., 2009). Metazoans can obtain these lipids from the diet or through *de novo* lipogenesis and cholesterogenesis. The sterol regulatory element-binding protein (SREBP) family of transcription factors play critical and conserved roles in regulating the transcription of enzymes required for lipogenesis and cholesterogenesis (Brown and Goldstein, 1997; Jeon and Osborne, 2012; Osborne and Espenshade, 2009; Rawson, 2003; Shimano and Sato, 2017).

In mammals, there are three SREBP family members: SREBP-1A, SREBP-1C and SREBP-2. SREBP-1A and SREBP-1C are produced from alternative splicing of the sterol regulatory element binding transcription factor 1 (*SREBF1*) transcripts, with SREBP-1A possessing an additional 24 amino acid residues in its transactivation domain, thus displaying stronger transcriptional activity than SREBP-1C (Goldstein et al., 2002; Shimano and Sato, 2017). SREBP-1C is broadly expressed in most tissues and plays a major role in nutritional regulation of the expression of lipogenic enzymes, such as acetyl-CoA carboxylase (ACC; the rate-limiting enzyme for *de novo* lipogenesis; also known as ACAC in mammals), fatty acid synthase (FASN; the key enzyme for lipogenesis) and acetyl coenzyme A synthase (AcCoAS; also known as ACSS in mammals) (Amemiya-Kudo et al., 2002; Desvergne et al., 2006; Shimano et al., 1999; Shimomura et al., 1999). In contrast, SREBP-1A is predominantly expressed in certain tissues and most cultured cells and regulates the expression of genes encoding both lipogenic and cholesterogenic enzymes. SREBP-2, encoded by the *SREBF2* gene, regulates the expression of factors involved in cholesterol metabolism (Goldstein et al., 2002; Shimano and Sato, 2017). SREBP transcription factors are unusual in that full-length SREBPs are localized on the membrane of the endoplasmic reticulum (ER). These full-length SREBPs are precursors consisting of a bHLH-Zip DNA-binding domain at the N-terminus, a transmembrane domain, and a regulatory domain at the C-terminus (Osborne and Espenshade, 2009). In the presence of sterols (or unsaturated fatty acids in the case of SREBP-1 processing), full-length SREBPs associate with two integral membrane proteins – SREBP cleavage-activating protein (SCAP) and insulin-induced gene (INSIG) – in the ER. When the intracellular level of sterols or fatty acids is low, INSIG is released and SREBP-SCAP is transported to the Golgi apparatus, where the full-length SREBP precursors are cleaved by site-1 protease (S1P; also known as MBTPS1 in mammals) and site-2 protease (S2P; also known as MBTPS2 in mammals), resulting in the release of the N-terminal fragment with the bHLH-Zip DNA-binding domain (Goldstein et al., 2002; Rawson, 2003; Wu et al., 2022). These N-terminal SREBP fragments enter the nucleus and stimulate the expression of SREBP target genes. Accordingly, these mature forms of SREBPs are also known as nuclear SREBPs (nSREBPs; unless otherwise specified, this work focuses on the mature or nSREBPs). This model provides an elegant explanation of how SREBPs maintain the homeostasis of intracellular sterols and fatty acids (Goldstein et al., 2002; Shimano and Sato, 2017), but the molecular mechanism that modulates the transcriptional activities of SREBPs in the nucleus is still not fully understood.

Consistent with the key role of SREBPs in regulating the transcription of lipogenic and cholesterogenic factors, dysregulation of SREBPs is found in metabolic disorders and various human cancers (Guo et al., 2014; Moslehi and Hamidi-Zad, 2018). Given that elevated fatty acid biosynthesis and increased expression of lipogenic enzymes such as FASN and ACC have been identified as a near-universal feature of human cancers, therapeutic approaches have been developed to target the key lipogenic enzymes, such as ACC, FASN and stearoyl-CoA desaturase 1 (Baenke et al., 2013; Cheng et al., 2018; Guo et al., 2014; Menendez and Lupu, 2007; Shimano and Sato, 2017). Understanding the function and regulation of SREBPs may provide mechanistic insights into these diseases, thereby aiding developing new approaches to treatment.

Although the components of the SREBP pathway are conserved in metazoans, the regulatory mechanisms are much simpler in invertebrates such as *Drosophila* and *Caenorhabditis elegans*. There is only one SREBP ortholog in *Drosophila*, in addition to single orthologs of other factors that control SREBP processing, including SCAP, S1P and S2P (Goldstein et al., 2002; Rawson, 2003). Instead of being inhibited by intracellular sterols, the proteolytic procession of SREBP is regulated by phosphatidylethanolamine (Dobrosotskaya et al., 2002; Seegmiller et al., 2002), consistent with the notion that arthropods and nematodes are auxotrophic for sterols due to the lack of critical cholesterogenic enzymes (Carvalho et al., 2010; Rawson et al., 1999; Rosenfeld and Osborne, 1998). Interestingly, unsaturated fatty acids such as oleate, linoleate and arachidodate inhibited the procession of SREBP-1A and SREBP-1C, but not that of SREBP-2, in cultured human embryonic kidney (HEK) 293 cells (Hannah et al., 2001). Together with the additional evidence summarized below, these discoveries suggest that *Drosophila* SREBP plays a similar role to mammalian SREBP-1C in regulating *de novo* lipogenesis.

**Fig. 1.**
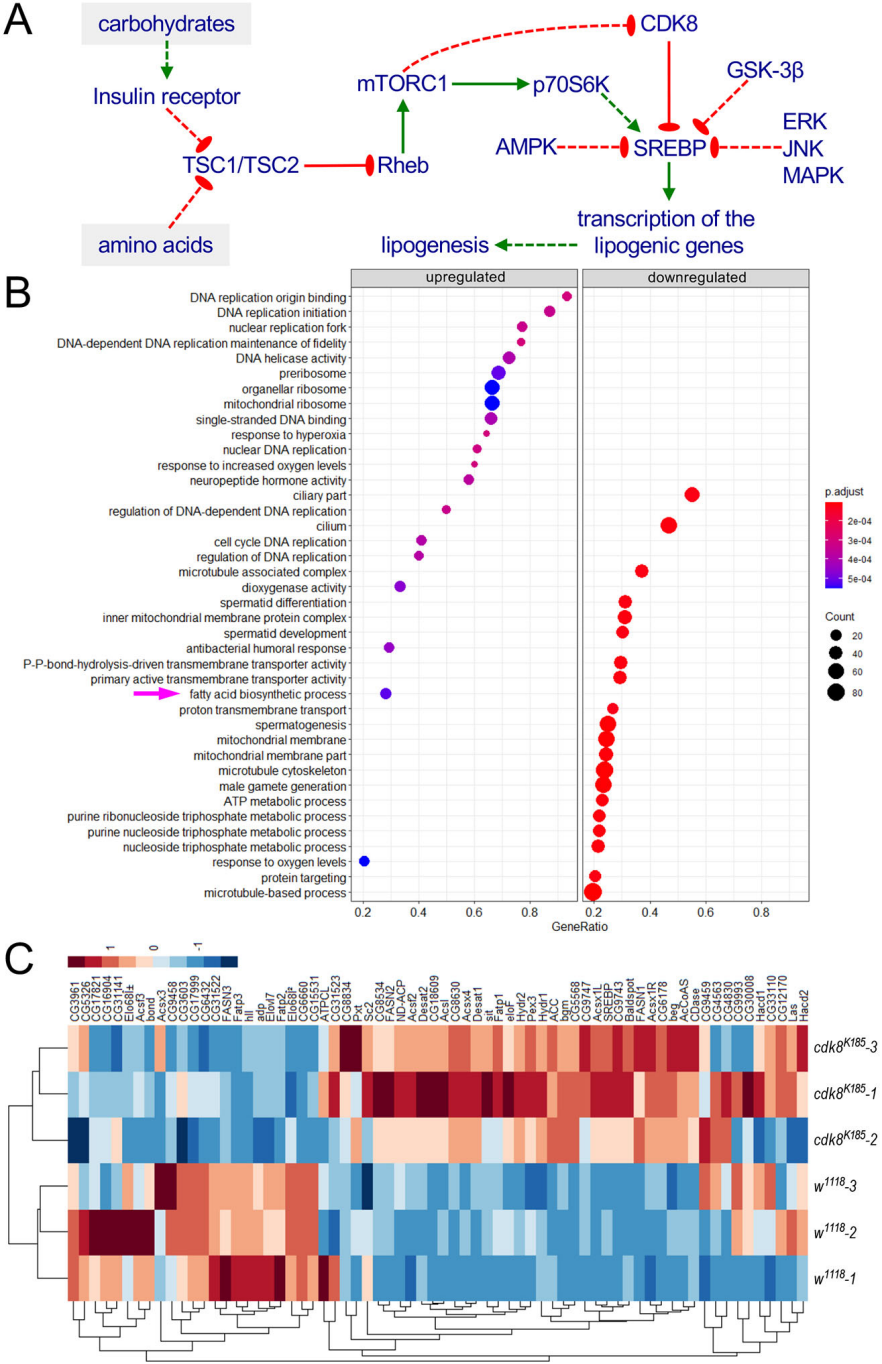
**Elevated lipogenic gene expression in *Cdk8* null mutants.** (A) Model: the role of Cdk8 and other protein kinases in mediating the dietary effects of carbohydrates and amino acids on SREBP and lipogenesis through the insulin/mTOR signaling pathway (also see Results and Discussion sections for details). (B) Dot plot of the top 40 Gene Ontology categories of significantly altered genes in *Cdk8^K185^* mutant larvae compared with the control (*w^1118^*) larvae at the third-instar wandering stage. (C) Heatmap of genes involved in the fatty acid biosynthetic process category of triplicate samples of *w^1118^* (control) and *Cdk8^K185^* mutant larvae at the third-instar wandering stage.

*De novo* lipogenesis is under tight regulation of physiological conditions such as feeding and fasting. Extensive studies have established that the insulin/mechanistic target of rapamycin (mTOR) signaling pathway is activated by dietary nutrients, such as carbohydrates and amino acids (Edgar, 2006; Jewell et al., 2013; Sabatini, 2017). Interestingly, downregulation of CDK8-CycC by insulin signaling is dependent on (mTOR complex 1) mTORC1 in cultured mammalian cells (Feng et al., 2015), while depleting Cdk8 or CycC abolishes the effects of mTOR activation on autophagosome formation in *Drosophila* (Tang et al., 2018). Moreover, *Cdk8* and *CycC* mutant larvae are hypersensitive to high-sugar diets and high levels of certain dietary amino acids that stimulate Tor in *Drosophila* (Gao et al., 2018). Collectively, these results suggest that mTORC1 functions upstream of Cdk8-CycC (Fig. 1A) (Li et al., 2019).

We have previously reported that CDK8 and its regulatory partner cyclin C (CycC; also known as CCNC in mammals) negatively regulate lipogenesis by inhibiting the transcriptional activity of the nSREBPs in both *Drosophila* and mammals (Zhao et al., 2012). CDK8 and CycC are two conserved subunits of the Mediator complex, a transcription co-factor complex required for RNA polymerase II-dependent transcription. CDK8 and CycC, together with MED12 and MED13, form the CDK8 kinase module (CKM). The CKM binds to the small Mediator complex, which is composed of ∼26 subunits conserved in eukaryotes, and forms the large Mediator complex (Allen and Taatjes, 2015; Dannappel et al., 2018; Soutourina, 2018; Yin and Wang, 2014). Specifically, reduction of CDK8 and CycC increases the transcription of lipogenic genes and fat accumulation in *Drosophila*, cultured mammalian cells and mouse livers (Zhao et al., 2012). Using *in vitro* kinase assays, we previously identified a conserved threonine residue, Thr402 of human SREBP-1C (or Thr390 of *Drosophila* SREBP), as the phosphorylation site by CDK8 (Zhao et al., 2012). Moreover, we showed that depleting CDK8 or CycC in cultured HEK293 cells reduces the levels of ubiquitinated nSREBP-1A, but increases the total protein level of nSREBP-1A, suggesting that phosphorylation of nSREBPs by CDK8 destabilizes nSREBP through ubiquitin-dependent protein degradation (Zhao et al., 2012). However, the exact mechanisms of how CDK8 interacts with SREBP and the biological consequences of SREBP phosphorylation by CDK8 *in vivo* are still not fully understood*.*

Here, we further investigated the role of phosphodeficient SREBP (nuclear form of *Drosophila* SREBP-Thr390Ala, designated as the SREBP^T390A^ or SREBP^TA^ mutant) in regulating its stability and transcriptional activities in *Drosophila*. In comparison to wild-type nSREBP, SREBP^T390A^ mutant proteins are more stable and more potent in stimulating the transcription of SREBP target genes and fat accumulation *in vivo*. Further mapping of the interactions between Cdk8 and SREBP led to the identification of six amino acids at the N-terminus of SREBP that are essential for the direct interaction between SREBP and Cdk8. Interestingly, these six amino acids, conserved from flies to humans, are also required for the direct interactions between SREBP and the MED15 subunit of the small Mediator complex. Collectively, these results support a key role of Cdk8-meditated SREBP phosphorylation in regulating lipogenesis *in vivo*, suggesting that the concerted interplay between Cdk8 and MED15 is important for the tight control of the transcriptional activities of nSREBP.

## RESULTS

### Validation of the role of Cdk8 in regulating lipogenic gene expression using RNA-seq analyses

In previous work, we characterized the effects of Cdk8 and CycC mutations on gene expression using microarray analyses of *Cdk8^K185^* and *CycC^Y5^* null mutant larvae (Zhao et al., 2012). However, this method holds several drawbacks such as high background owing to cross-hybridization, limited dynamic range of detection due to saturated signals, and limited specificity and sensitivity, especially for low-abundance transcripts (Jaluria et al., 2007). To combat the disadvantages of microarray analysis, we carried out transcriptome profiling of the *Cdk8^K185^* homozygous larvae using RNA-sequencing (RNA-seq) analyses (see Materials and Methods for details). In comparison to the control group, 4034 genes were significantly upregulated, and 2867 genes were significantly downregulated, in *Cdk8^K185^* mutants. These genes were categorized using the Gene Set Enrichment Analysis of Gene Ontology (GseGO) function of the clusterProfiler package (Yu et al., 2012). The top 40 Gene Ontology categories are shown as a dot plot for *Cdk8^K185^* (Fig. 1B). The fatty acid biosynthesis process was identified as one of the most significantly upregulated gene categories among these top 40 Gene Ontology categories (Fig. 1B), consistent with our previous report of elevated levels of fatty acid biosynthesis in *Cdk8^K185^* mutant larvae (Zhao et al., 2012).

To display the changes in genes involved in the fatty acid biosynthesis process at high resolution, we used a heatmap to show all the genes within the category of biological triplicates of *Cdk8^K185^* and the control (*w^1118^*). The triplicates of the same genotypes were clustered together according to the *x*-axis clustering (Fig. 1C), which validates correct sampling of these biological replicates. Importantly, transcription of several key lipogenic genes, such as *FASN1*, *FASN2*, *ACC* and *AcCoAS*, is significantly elevated in *Cdk8^K185^* mutant larvae. These lipogenic genes are known direct transcriptional targets of SREBP, consistent with our previous report that SREBP is negatively regulated by CDK8 (Zhao et al., 2012). The significantly changed genes were then mapped into the fatty acid synthesis pathway with the Pathview package (Luo and Brouwer, 2013). Although not all the genes related to lipid biosynthesis were increased, genes encoding key lipogenic enzymes, marked in red in Fig. S1, were significantly increased. Thus, these RNA-seq analyses provide further support for the inhibitory effects of Cdk8 on SREBP-dependent lipogenic gene expression.

### Increased stability of phosphodeficient SREBP proteins *in vivo*

**Fig. 2.**
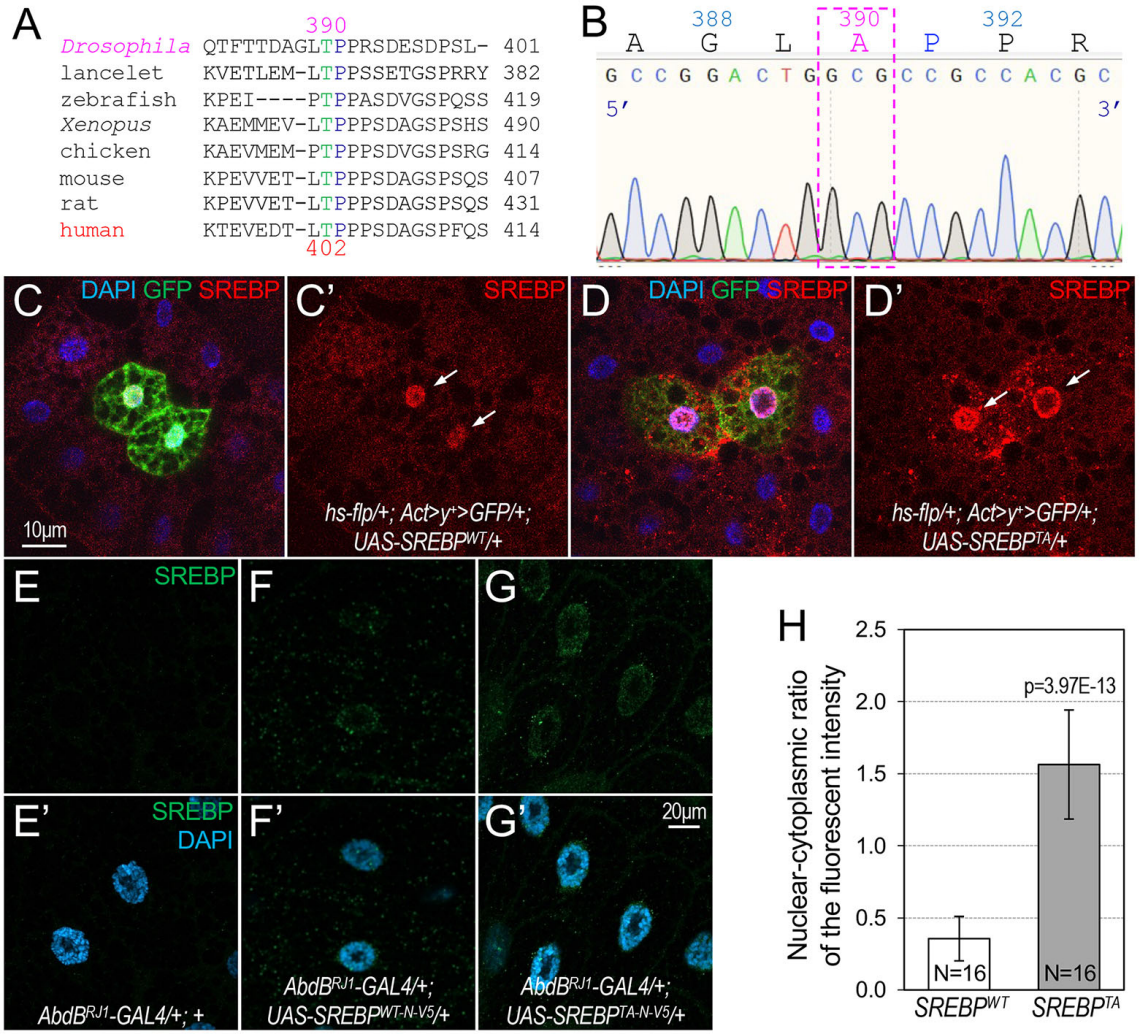
**Increased stability of the phosphodeficient SREBP proteins.** (A) Alignment of the amino acid sequences of SREBP to show the conservation of Thr390Pro391 (TP) from *Drosophila* to humans. (B) Validation of *Drosophila* SREBP-Thr390Ala mutation by sequencing*.* (C,D) Representative confocal images of larval fat body stained with an anti-SREBP antibody (red), GFP (green) and the DNA dye DAPI (blue). Detailed genotypes are *hs-flp/+; Actin>y^+^>GFP/+; UAS-SREBP*^WT^/+ (C); *hs-flp/+; Actin>y^+^>GFP/+; UAS-SREBP^TA^/+* (D). (C′,D′) The same images as in C,D with only the anti-SREBP staining (red) shown*.* Scale bar: 10 μm. (E-G′) Representative confocal images of immunostaining with an anti-SREBP antibody (green; E-G′) and DAPI (blue; E′,F′,G′) in salivary glands from *AbdB^RJ1^-Gal4/+; UAS-SREBP*^WT^/+ (E,E′) and *AbdB^RJ1^-Gal4/+; UAS-SREBP^TA^/+* (F-G′) larvae. Merged images are shown in E′-G′. Scale bar: 20 μm. (H) Quantification of the nuclear-cytoplasmic ratio of the fluorescent intensity of anti-V5 antibody staining in salivary gland cells with ectopic expression of V5-tagged SREBP^WT^ or SREBP^TA^ (SREBP^T390A^) using ImageJ (*n*=16 for the indicated genotypes). The *P*-value was calculated based on one-tailed unpaired *t*-tests, and error bars indicate s.d.

Our previous *in vitro* biochemical analyses suggest that CDK8 may inhibit lipogenesis by directly phosphorylating SREBP at a highly conserved threonine residue, Thr390 in *Drosophila* SREBP or Thr402 in human SREBP-1C (Fig. 2A) (Zhao et al., 2012). To test whether SREBP phosphorylation by Cdk8 regulates the stability and the transcriptional activity of SREBP *in vivo*, we analyzed the effects of mutating the Thr390 residue of SREBP on those properties of SREBP in *Drosophila*. Specifically, we generated two transgenic lines to ectopically express the nuclear form [amino acids (AA)1-451] of either wild-type SREBP (*UAS-SREBP*^WT^) or the phosphodeficient SREBP (*UAS-SREBP^T390A^*). The T390A mutation was validated by sequencing as shown in Fig. 2B. To reduce potential variations caused by the chromatin environments of the insertion sites, we used the *pVALIUM10-roe* vector, which flanks the expression cassettes with *gyspy* insulators and supports site direct insertion with *attB* integrase target sequence (Ni et al., 2009; Perkins et al., 2015). The constructs were then inserted in the *attP2* genomic landing site on the third chromosome.

To test whether the stability of SREBP is affected in the phosphodeficient line, we performed a clonal analysis in the larval fat body using the *cis*-chromosomal recombination (FLP-out) system (Germani et al., 2018). Specifically, we constructed the larvae with the following genotypes: *hs-flp/+; Actin>y^+^>Gal4, UAS-GFP/+; UAS-SREBP*^WT^/+ and *hs-flp/+; Actin>y^+^>Gal4, UAS-GFP/+; UAS-SREBP^T390A^/+.* GFP-labeled adipocytes overexpress either wild-type SREBP (SREBP^WT^) or SREBP^T390A^ mutant proteins, and the expression level of SREBP was detected by immunostaining using an anti-SREBP polyclonal antibody (Xie et al., 2015). Compared to the surrounding cells, ectopic expression of either form of SREBP in the GFP-positive adipocytes significantly increased the SREBP levels as expected (Fig. 2C,D). Importantly, a higher level of SREBP^T390A^ proteins compared to wild-type SREBP was observed in these clones (Fig. 2C′ versus D′), suggesting that phosphodeficient SREBP proteins are more stable than wild-type SREBP proteins *in vivo*.

To further test the effects of phosphodeficient SREBP on its stability, we ectopically expressed either wild-type or phosphodeficient SREBP proteins in the salary glands of third-instar larvae and then analyzed the levels of SREBP. Overexpression of either SREBP^WT^ or SREBP^T390A^ proteins in the salivary glands using *Sgs3-Gal4* and *C96-Gal4* causes lethality in the early larval stage (M.Z., X.L., and J.-Y.J., unpublished observations); thus, we used a weak salivary gland-specific Gal4 line, *AbdB^RJ1^-Gal4*, which we generated in an unrelated project (R.-U.-S.H.-W. and J.-Y.J., unpublished data). This Gal4 line is weaker and more specific than other Gal4 lines that are active in the salivary glands, such as *Sgs3-Gal4* and *C96-Gal4*. In addition, we generated a new set of transgenic lines by modifying the *pVALIUM10-roe* vector with 5xUAS, instead of the original 10xUAS (Ni et al., 2009; Perkins et al., 2015). To facilitate the detections, we also added a V5 epitope tag to the N-terminus of SREBP^WT^ or SREBP^T390A^. The constructs were both targeted into the same *attP2* loci to ensure the same genomic environment. These genetic tools allowed us to achieve low, but reliable, ectopic expression of SREBP proteins in salivary gland cells. As shown in Fig. 2F,F′, we detected weak SREBP protein expression in salivary glands cells, compared to the control, in which little anti-V5 signal was detected (Fig. 2E,E′). Compared to the salivary glands with ectopic expression of SREBP^WT^-V5, we observed significantly stronger anti-V5 antibody staining in the nuclei of salivary glands with ectopic expression of SREBP^T390A^-V5 (Fig. 2G-G″). These effects were quantified by measuring the nuclear-cytoplasmic ratio of the fluorescent intensities of the salivary gland cells (Fig. 2H). These observations indicate that phosphodeficient SREBP proteins are more stable than wild-type SREBP proteins.

We previously proposed a model to explain how CDK8-CycC inhibits SREBP-dependent gene expression, which posits that SREBP phosphorylation by CDK8 promotes the degradation of SREBP (Zhao et al., 2012). Consistent with this model, SREBP phosphorylation by Cdk8 increased the ubiquitination and degradation of SREBP (Zhao et al., 2012), and the E3 ligase SCF^Fbw7b^ is required for ubiquitination of SREBP (Sundqvist et al., 2005). Our new observations are consistent with the stabilization of the corresponding Thr402 to alanine mutation in human SREBP-1C (Zhao et al., 2012).

### Effects of phosphodeficient SREBP in stimulating lipogenic gene expression *in vivo*

**Fig. 3.**
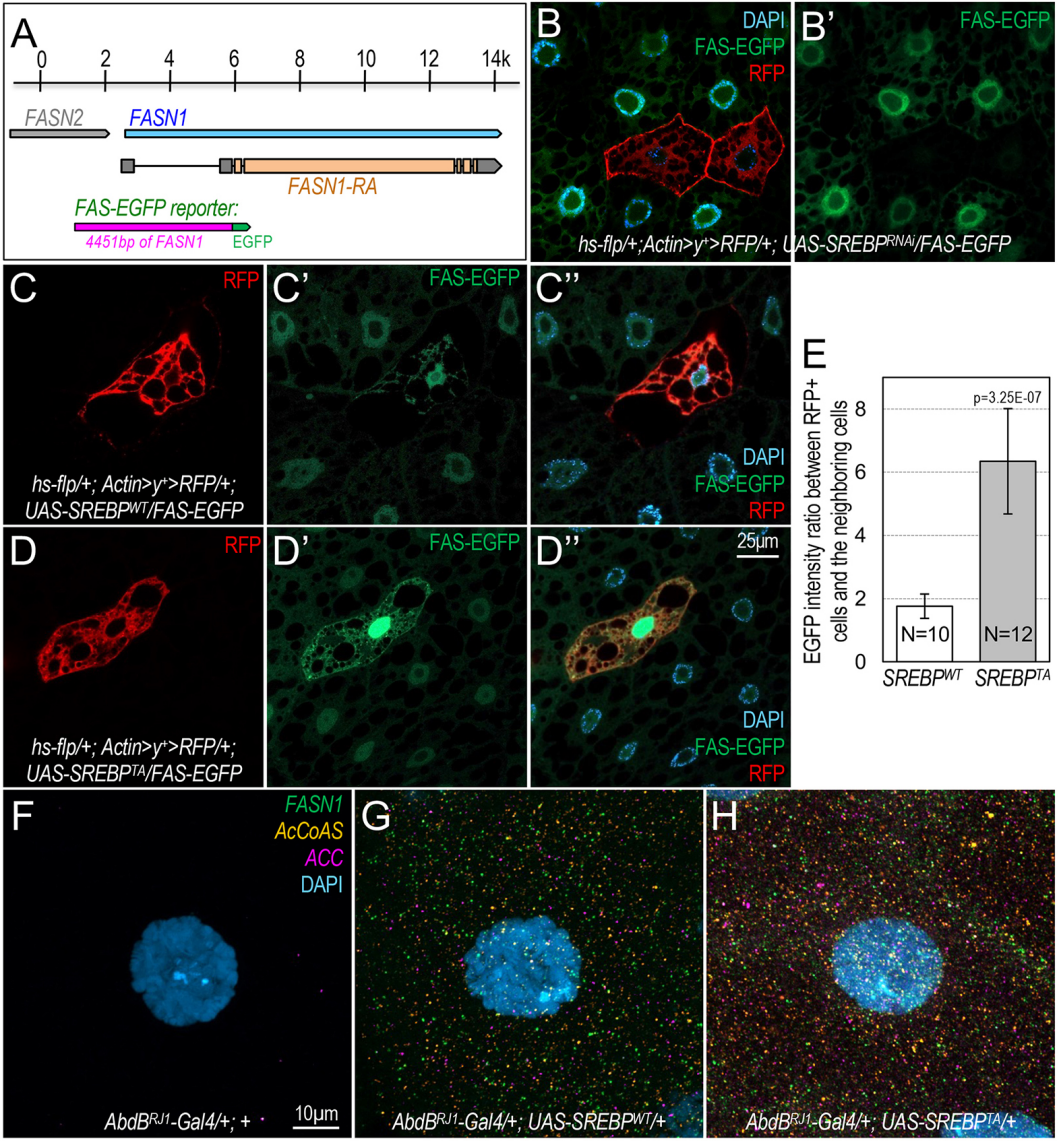
**Effects of phosphodeficient SREBP in stimulating lipogenic gene expression *in vivo*.** (A) Scheme of the *FAS-EGFP* reporter: a 4451 bp fragment upstream of the *FASN1* gene from a BAC clone was used to drive the expression of an EGFP reporter. (B,B′) Confocal images of FAS-EGFP (green), RFP (red) and DAPI (blue) in *Drosophila* larval fat body of the *hs-flp/+; Actin>y^+^>RFP/+; UAS-SREBP^RNAi^/FAS-EGFP* genotype; only FAS-EGFP (green) is shown in B′*.* (C-D″) Representative confocal images of RFP (red; C,D), FAS-EGFP (green; C′,D′), and DAPI (blue; merged images shown in C″,D″) in larval fat body of the following genotypes: *hs-flp/+; Actin>y^+^>RFP/+; UAS-SREBP^WT^/FAS-EGFP* (C); *hs-flp/+; Actin>y^+^>RFP/+; UAS-SREBP^T390A^/FAS-EGFP* (D)*.* Scale bar: 25 μm. (E) Quantification of the EGFP levels in cells marked with RFP, normalized to the EGFP levels in neighboring non-RFP cells using ImageJ (*n*=10-12). The *P*-value was calculated based on one-tailed unpaired *t*-tests, and error bars indicate s.d. (F-H) Detection of SREBP target gene expression in larval salivary glands of the indicated genotypes by *in situ* hybridization chain reaction (HCR) assay. *FASN1* (green), *AcCoAS* (orange) and *ACC* (magenta) probe sets were used with B1-Alexa Fluor 488, B2-Alexa Fluor 594 and B3-Alexa Fluor 647 amplifiers, respectively, in multiplexed *in situ* HCR. These images are projections of 12 successive optical sections (1 μm apart). Scale bar: 10 μm.

To test whether increased stability of SREBP^T390A^ correlates with more potent transcriptional activity at the cellular level, we generated a transgenic reporter line using a 4.4 kb promoter region of the *Drosophila* fatty acid synthase gene *FASN1* to drive the expression of EGFP (Fig. 3A), designated as the *FAS-EGFP* reporter. We then genetically recombined this reporter with the Gal4/UAS flip-out system (*Actin>y^+^>Gal4 UAS-RFP*). Clonal depletion of SREBP in adipocytes, as marked with RFP, resulted in reduced EGFP levels compared with those of their neighboring cells (Fig. 3B,B′), suggesting that SREBP is required for the expression of this *FAS-EGFP* reporter. Conversely, overexpression of either SREBP^WT^ or SREBP^T390A^ significantly increased the levels of EGFP (Fig. 3C-D″), suggesting that SREBP is sufficient to drive expression of the *FAS-EGFP* reporter. Importantly, SREBP^T390A^ proteins are more potent than SREBP^WT^ in stimulating *FAS-EGFP* expression (compare Fig. 3D′ and C′), and the results from multiple clones are quantified and shown in Fig. 3E.

To further validate the effects of different forms of SREBP on its target gene transcription in a different tissue, we analyzed the expression of *FANS1*, *AcCoAS* and *ACC* in larval salivary glands. To directly visualize the transcripts of these genes, we used *in situ* hybridization chain reaction (HCR) RNA fluorescence *in situ* hybridization (RNA-FISH) imaging technology, a sensitive, quantitative and robust method that allows simultaneous imaging of specific mRNA transcripts at the cellular level (Choi et al., 2010, 2018; Dirks and Pierce, 2004). As expected, ectopic expression of SREBP^WT^ in salivary glands stimulated the expression of these SREBP target genes (compare Fig. 3G with the control shown in F). Compared to SREBP^WT^, many more mRNA products of these genes were observed when SREBP^T390A^ was expressed (Fig. 3H; Fig. S2). Taken together, these observations show that the phosphodeficient SREBP is more potent than SREBP^WT^ in activating the transcription of SREBP target genes.

### Effects of phosphodeficient SREBP in stimulating lipid accumulation

**Fig. 4.**
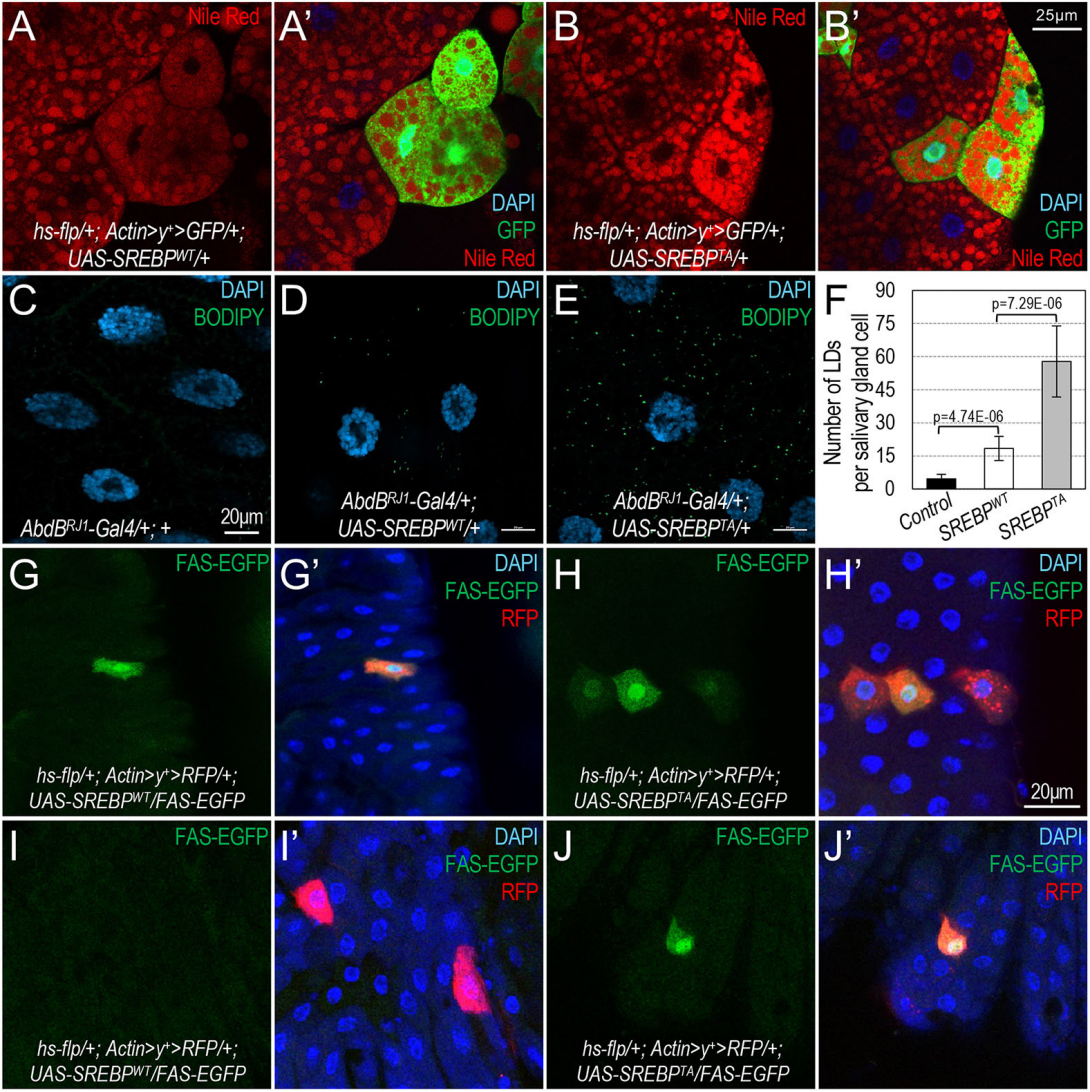
**Effects of phosphodeficient SREBP in stimulating lipid accumulation.** (A-B′) Representative confocal images of larval fat body stained with Nile Red (red), GFP (green) and DAPI (blue) of the following genotypes: *hs-flp/+; Actin>y^+^>RFP/+; UAS-SREBP*^WT^/*FAS-EGFP* (A,A′); *hs-flp/+; Actin>y^+^>RFP/+; UAS-SREBP^T390A^/FAS-EGFP* (B,B′)*.* The signals of Nile Red staining of the same images are shown in A and B. Scale bar: 25 μm. (C-E) Confocal images of salivary glands from the third-instar wandering larvae of the following genotypes: *AbdB^RJ1^-Gal4/+* (C); *AbdB^RJ1^-Gal4/+; UAS-SREBP*^WT^/+ (D); and *AbdB^RJ1^-Gal4/+; UAS-SREBP^T390A^/+* (E)*.* These salivary glands were stained with BODIPY (green) and DAPI. Scale bars: 20 μm. (F) The average number of lipid droplets (LDs) per salivary gland cells from larvae of the indicated genotypes (C-E). *n*=10 for each genotype; *P*-values were calculated using one-tailed unpaired *t*-tests, and error bars indicate s.d. (G-J′) Confocal images of FAS-EGFP (green), RFP (red) and DAPI (blue) in *Drosophila* adult midgut of the following genotypes: *hs-flp/+; Actin>y^+^>RFP/+; UAS-SREBP*^WT^/*FAS-EGFP* (G-I′); *hs-flp/+; Actin>y^+^>RFP/+; UAS-SREBP^T390A^/FAS-EGFP* (H-J′)*.* The effects of feeding (G,H) and starvation (I,J) were analyzed. FAS-EGFP of the same images is shown in G,I and H,J; merged images are shown in G′,I′ and H′,J′. Scale bar: 20 μm.

To investigate the function of phosphodeficient SREBP in promoting lipogenesis, we used a similar FLP-out system to generate somatic clones in the larval fat body, marked by GFP, to overexpress either SREBP^WT^ or SREBP^T390A^, and then analyzed lipid accumulation by staining lipid droplets using Nile Red. As shown in Fig. 4A,A′, clonal expression of SREBP^WT^ in cells marked with GFP had no obvious effects on lipid droplets compared to their neighboring adipocytes, presumably because mature SREBP proteins are maximally activated in adipocytes at the wandering larval stage, or the ectopically expressed SREBP^WT^ proteins are actively degraded. In contrast, overexpressing phosphodeficient SREBP^T390A^ resulted in stronger Nile Red staining in adipocytes marked with GFP, compared to their neighboring adipocytes (Fig. 4B,B′).

The abundant lipid droplets accumulated in adipocytes of the late-third-instar larvae resulted in a high baseline level of lipid staining, making it challenging to make a quantitative comparison between the two forms of SREBP protein based on lipid accumulation. Thus, we tested these effects in salivary gland cells, which normally accumulate few or no detectable lipid droplets (Fig. 4C). Ectopic expression of SREBP^T390A^ in the salivary gland using *AbdB^RJ1^-Gal4* to overexpress SREBP^WT^ significantly increased the number of lipid droplets (Fig. 4D, quantification is shown in F). Compared to SREBP^WT^, we observed significantly more lipid droplets per cell when SREBP^T390A^ was ectopically expressed using the same approach (Fig. 4E,F). Collectively, these results show that phosphodeficient SREBP^T390A^ proteins are more potent at stimulating lipogenic gene expression and lipid accumulation than the wild-type SREBP *in vivo*.

To further validate the effects of different forms of SREBP on *FASN1* transcription in a biological context, we carried out a similar experiment in the adult intestine and analyzed the effects of starvation. Starvation is expected to inhibit mTOR, which correlates with elevated Cdk8 protein levels in *Drosophila* larvae (Xie et al., 2015). This model predicts that elevated Cdk8 phosphorylates and stimulates the degradation of nSREBP^WT^, but not phosphodeficient SREBP^T390A^. If so, only SREBP^T390A^ is expected to stimulate the expression of the *FAS-EGFP* reporter under starvation conditions (Fig. 1A). To test this prediction and to avoid potential positional effects in the midgut for our comparison, we focused our analyses on a posterior region of the midgut, near the midgut and hindgut boundary, in adult female flies. The expression of *FAS-EGFP* in cells within this region was low (Fig. 4G). As expected, overexpression of either SREBP^WT^ or SREBP^T390A^ in cells marked with RFP (Fig. 4G′,H′) stimulated the expression of the *FAS-EGFP* reporter under a normal feeding condition (Fig. 4G,H). However, under starvation conditions, ectopic expression of SREBP^WT^ in cells marked with RFP (Fig. 4I′) failed to stimulate *FAS-EGFP* expression; its level was similar to that in the neighboring cells (Fig. 4I). In contrast, ectopically expressing the phosphodeficient SREBP^T390A^ (the cell marked with RFP, Fig. 4J′) can still stimulate the expression of the *FAS-EGFP* reporter compared to that in neighboring cells (Fig. 4J). These observations suggest that both SREBP^WT^ and phosphodeficient SREBP^T390A^ can stimulate lipogenic gene transcription *in vivo*, but only SREBP^T390A^ is resistant to starvation in this process. These results further support the key function of Cdk8-dependent phosphorylation of SREBP in the context of nutritional regulation of *de novo* lipogenesis (Fig. 1A).

### The N-terminus of SREBP directly interacts with Cdk8

The N-terminus of human SREBP-1A (AA30-40) fused with GST can pull down several Mediator subunits, including CDK8, MED1, MED6 and MED15, from the HeLa cell nuclear extract (Yang et al., 2006). However, it is unclear whether Cdk8 directly interacts with SREBP, or indirectly through the small Mediator complex, and whether the physical interactions between SREBP and MED15 are conserved in *Drosophila*. To test whether Cdk8 directly interacts with SREBP and to further map the specific regions mediating this interaction, we performed three rounds of GST pull-down analyses.

**Fig. 5.**
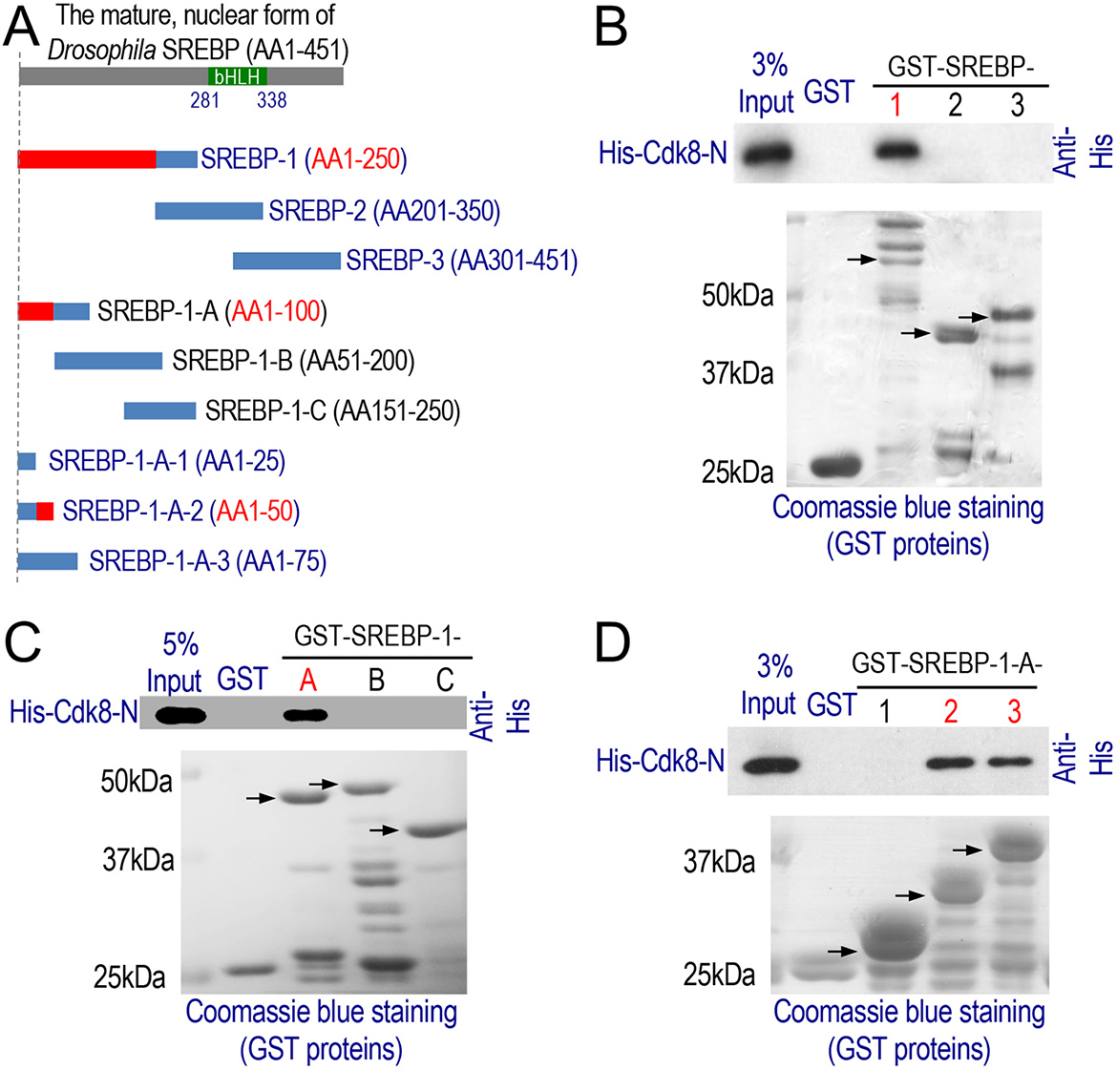
**Direct interactions between Cdk8 and nuclear SREBP.** (A) Scheme of the nuclear form of *Drosophila* SREBP, containing a bHLH domain from AA281-338, as well as different SREBP fragments that were used to generate GST-fusion proteins. (B-D) Western blots showing the three rounds of GST pull-down assays that were used to further map the specific regions in SREBP that can directly interact with His-tagged Cdk8. AA, amino acids.

Specifically, we generated polyhistidine (His)-tagged Cdk8 (AA1-262), containing the ATP-binding site, A-loop, CycC interface and substrate-binding domain (Schneider et al., 2011; Xu et al., 2014). In addition, we generated GST-fusion proteins by dividing SREBP into three partially overlapping fragments (Fig. 5A), designated as GST-SREBP-1 (AA1-250), GST-SREBP-2 (AA201-350, containing the bHLH-Zip domain) and GST-SREBP-3 (AA301-451), respectively. As shown in Fig. 5B, His-Cdk8-N (AA1-262) interacted with GST-SREBP-1, but not GST-SREBP-2 or GST-SREBP-3, suggesting that AA1-200 of SREBP interacts with Cdk8 *in vitro*. Next, we generated three smaller and partially overlapping fragments of GST-SREBP-1 and carried out a similar GST pull-down assay. Only GST-SREBP-1-A (AA1-100; Fig. 5A), but not GST-SREBP-1-B (AA51-200) or GST-SREBP-1-C (AA151-250), interacted with His-Cdk8-N (Fig. 5C), suggesting that AA1-50 of SREBP directly binds to Cdk8. In our third round of mapping, we observed that GST-SREBP-1-A-2 (AA1-50) and GST-SREBP-1-A-3 (AA1-75), but not GST-SREBP-1-A-1 (AA1-25), interacted with His-Cdk8-N (Fig. 5A,D). These observations show that the AA25-50 at the N-terminus of SREBP directly interacts with Cdk8 *in vitro*, which is consistent with the notion that SREBP protein is a direct target of Cdk8.

### The N-terminus of SREBP directly interacts with MED15

**Fig. 6.**
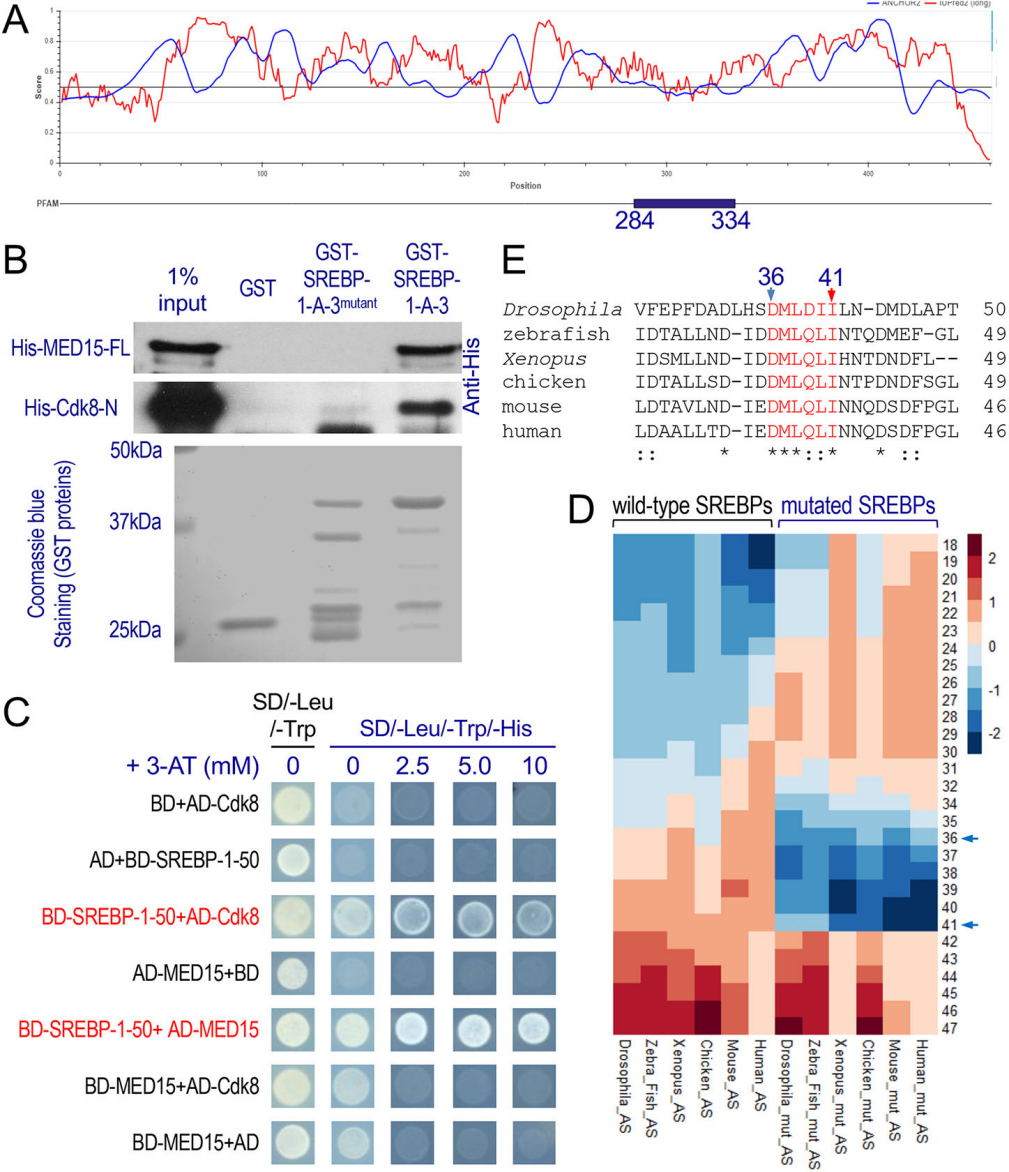
**Interactions between the N-terminus of SREBP and Cdk8 or MED15.** (A) Prediction of disordered binding regions in the mature SREBP using ANCHOR2 (blue) and disordered protein regions using IUPred2 (red). (B) Western blots of the GST pull-down assay: interactions between the wild-type or AA36-41 mutated to alanine residues in GST-SREBP-1-A-3 fragments and His-tagged N-terminal half of Cdk8 or full-length MED15. (C) Yeast two-hybrid assay showing the interaction between SREBP AA1-50 with either Cdk8 (full length) or MED15 (full length). SD/−Leu/−Trp and SD/−Leu/−Trp/−His are synthetic dropout (SD) media lacking Leu and Trp, or Leu, Trp and His, respectively. AD, GAL4-activation domain (prey); BD, GAL4-DNA-binding domain (bait); 3-AT, 3-amino-1,2,4-triazole (at 2.5, 5.0 or 10 mM, which competitively inhibits the *HIS3* gene product in budding yest, thereby increasing the stringency of the assay). (D) Heatmap of ANCHOR2 predication of disordered binding regions of wild-type SREBPs (left half) or mutated SREBPs (‘mut’, shown in the right half) from different species. AS, ANCHOR2 score. (E) Sequence alignment of the N-terminus of *Drosophila* SREBP (AA25-50) with SREBP proteins from other species. The highly conserved six amino acids (AA36-41) are highlighted in red. Asterisks (*) indicate residues that are entirely conserved in all species; colons (:) indicate residues that are highly conserved in all species, scoring >0.5 in the Gonnet PAM 250 matrix.

The nSREBP contains a structured bHLH-Zip DNA-binding domain (AA284-334), but other parts of the nSREBP are largely intrinsically disordered (Fig. 6A). This is perhaps a reason why only the structure of the DNA-binding domain of human nSREBP-1A has been resolved using X-ray crystallography (Parraga et al., 1998). Given that the AA30-40 region of human SREBP-1A can directly interact with the KIX domain of MED15 (Yang et al., 2006), we tested whether the interaction is conserved in *Drosophila*. As shown in Fig. 6B, GST-SREBP-1-A-3 (AA1-75) can also pull down His-tagged full-length *Drosophila* MED15 protein, suggesting that the interaction between the N-terminus of SREBP and MED15 is evolutionarily conserved. Using the yeast two-hybrid assay, we validated the interactions between SREBP AA1-50 and full-length Cdk8 or MED15 (Fig. 6C). Notably, these analyses suggest that the same region of SREBP (AA25-50) can directly interact with Cdk8 or MED15 (Fig. 5D, Fig. 6B,C). These interactions may have important implications in our understanding of the dynamic processes that involve the small Mediator complex and the CKM in SREBP-dependent gene expression (see Discussion).

To identify the specific amino acids within the N-terminus of SREBP that are required for direct interaction with CDK8 or MED15, we analyzed the intrinsically disordered regions of SREBP from different species. We used the ANCHOR2 program, which can robustly predict protein disorder based on an energy estimation approach, and can also detect regions that probably gain energy by interacting with globular proteins (Dosztanyi et al., 2009). As shown in Fig. 6D, we used a heatmap to visualize the ANCHOR2 scores (ASs) of the N-terminus of SREBP proteins from *Drosophila melanogaster*, zebrafish (*Danio rerio*; SREBP1), frog (*Xenopus laevis*; SREBP1), chicken (*Gallus gallus*; SREBP1), mouse (*Mus musculus*; SREBP-1A) and human (*Homo sapiens*; SREBP-1A). Interestingly, these analyses revealed a hot spot overlapping from AA35-44 across the species (Fig. 6D), suggesting that this region has higher potential to interact with other proteins. Within this region, six amino acids (DMLDII, AA36-41), are highly conserved during evolution (Fig. 6E).

To test whether mutating these six amino acids (DMLDII) can alter the protein-binding potential in this disordered region of the SREBP, we replaced them with six alanines from different species *in silico* and then analyzed the ASs of these mutant SREBPs using ANCHOR2, a web-based application developed for predicting disordered protein-binding regions (Dosztanyi et al., 2009). As shown in Fig. 6D, the AS hotspot is diminished within this region, indicating that these six conserved amino acids are important for the SREBP, Cdk8 and MED15 interactions. To verify this prediction, we mutated these six amino acids to six alanines in the GST-SREBP-1-A-3 (AA1-75) fragment, and then performed GST pull-down assays. As shown in Fig. 6B (lane 3), the mutation of these conserved six amino acids in GST-SREBP-1-A-3 abolished its interaction with both MED15 and Cdk8. These observations suggest that the conserved region within the N-terminus of SREBP, particularly the highly conserved but disordered region DMLDII (AA 36-41), is crucial in mediating the interactions between SREBP and Cdk8, or between SREBP and MED15.

## DISCUSSION

Previous studies have provided an elegant model to explain how SREBPs control intracellular fatty acid and cholesterol homeostasis. These studies mainly focus on understanding the mechanisms of how the full-length SREBP precursors are processed in the cytoplasm in response to intracellular levels of sterols and fatty acids (Brown and Goldstein, 1997; Jeon and Osborne, 2012; Osborne and Espenshade, 2009; Rawson, 2003; Shimano and Sato, 2017). However, exactly how the nSREBP proteins activate the transcription of lipogenic and cholesterogenic genes, and how this process is turned off in the nucleus are still not fully understood. In this study, we analyzed the *in vivo* effects of Cdk8 phosphorylation of SREBP on lipogenic gene transcription and *de novo* lipogenesis, and then further mapped the specific regions of SREBP that directly interact with Cdk8 and MED15 using *in vitro* and *in silico* approaches.

### Critical role of phosphorylation of SREBP Thr390 *in vivo*

Post-translational modifications of SREBPs, such as acetylation and phosphorylation, play critical roles in fine-tuning SREBP activities (Raghow et al., 2019; Zhao and Yang, 2012). Using *in vitro* kinase assays, we previously identified one specific phosphorylation site of SREBP by CDK8, i.e. Thr402 of human SREBP-1C, or Thr390 of *Drosophila* SREBP (Zhao et al., 2012). Further biochemical analyses using cultured mammalian cells suggest that Thr402 phosphorylation negatively regulates human SREBP-1C (Zhao et al., 2012). In this study, we investigated the biological consequences of this phosphorylation in *Drosophila*, particularly its effects on the stability of SREBP, as well as the activity of SREBP in stimulating target gene expression and lipid accumulation. Compared to the wild-type SREBP, SREBP^T390A^ phosphodeficient proteins were more stable, and were more potent in stimulating SREBP target gene expression and lipid accumulation *in vivo*. These results suggest that Cdk8 is a key kinase that restrains the transcriptional activity of SREBP in the nucleus (Fig. 1A).

Besides CDK8, previous studies using different experimental systems have shown that SREBPs can be phosphorylated at multiple threonine and serine residues by a number of protein kinases, including AMPK, GSK-3β, ribosomal protein S6 kinase (p70S6K), p38ERK, PKA and c-JUN N-terminal protein kinase (JNK) (Dong et al., 2015, 2014, 2020; Knebel et al., 2014; Li et al., 2011; Lu and Shyy, 2006; Punga et al., 2006). As discussed below, p70S6K phosphorylation of SREBP-1C positively regulates the processing of full-length SREBP-1C and stimulates nSREBP-1C activity (Dong et al., 2020), while phosphorylation by other kinases inhibits SREBP-1 activity (Fig. S3A). To consider the evolution implications of these mechanisms, we aligned the amino acid sequences of SREBP-1A and SREBP-1C homologs in a few representative species, including *Drosophila melanogaster*, zebrafish, *Xenopus*, chicken (*Gallus gallus*), mouse, rat and human. Of all the reported phosphorylation sites, the threonine residue phosphorylated by CDK8 (Fig. S3B), as well as several threonine/serine residues phosphorylated by GSK-3β and p70S6K (Fig. S3B,C), appear to be highly conserved from flies to humans. GSK-3β can phosphorylate human SREBP-1A at Thr426 (equivalent to Thr402 in human SREBP-1C) and Ser430 (Fig. S3B), thereby promoting ubiquitination and degradation of SREBP-1A (Punga et al., 2006; Sundqvist et al., 2005).

Similarly, rat SREBP-1C can be phosphorylated at Ser418, Ser419 and Ser422 residues upon insulin treatment (Fig. S3C), and p70S6K, a kinase downstream of insulin signaling, was shown to enhance SREBP-1C precursor maturation and protect SREBP-1C from proteasomal degradation (Dong et al., 2020). These phosphorylation sites or serine/threonine-rich regions seem to be conserved, indicating mechanistic conservation of SREBP phosphorylation by p70S6K in *Drosophila* and humans (Fig. S3C), which remains to be experimentally validated.

In contrast to Cdk8, GSK-3β and p70S6K, the phosphorylation sites of AMPK, p38ERK, PKA and JNK are not conserved in *Drosophila*. For example, it was reported that activation of AMPK by polyphenols and metformin can inhibit lipogenesis by phosphorylating SREBP-1C at Ser372, thereby suppressing SREBP-1C cleavage and nuclear translocation in mice and cultured human HepG2 hepatocytes (Li et al., 2011). The Ser372 residue of human SREBP-1C is conserved in lancelets and vertebrates, but the sequence in that region is not conserved in *Drosophila* (Fig. S3D). Similarly, Ser73 of rat SREBP-1C can be phosphorylated by GSK-3β, resulting in the dissociation of the SREBP-1C-SCAP complex and ubiquitination-dependent proteasomal degradation of SREBP-1C (Dong et al., 2015). However, the Ser73 residue appears to be only conserved in SREBP proteins of certain vertebrate species (Fig. S3E). In addition, Ser117 of human SREBP-1A can be phosphorylated by ERK and JNK, and mutating this phosphorylation site protects mice under normocaloric conditions from developing enlarged fatty livers (Kotzka et al., 2012), yet the Ser117 residue is also only conserved in mammals (Fig. S3F). Moreover, p38MAPK, ERK and JNK can also phosphorylate Ser39 and Thr402 of human SREBP-1C (Knebel et al., 2014). Taken together, these discoveries have revealed potential variations in the detailed molecular mechanisms of how insulin signaling stimulates SREBP-dependent lipogenic gene expression and promotes *de novo* lipogenesis during the evolution of metazoans. To our knowledge, the role of GSK-3β, NJK, p38ERK and p70S6K in regulating SREBP activity has not been tested in *Drosophila* and other invertebrates. Based on amino acid sequence conservation of SREBPs, phosphorylation of SREBP by CDK8, GSK-3β and p70S6K might be a more ancient regulatory mechanism than phosphorylation of SREBP by AMPK and MAPKs. Compared to in invertebrates, such as insects, additional kinases that regulate SREBP and lipogenesis may offer adaptive advantages in coping with more complex physiological needs that require fine-tuned control of the activities of the SREBP family of transcription factors in vertebrates or mammals.

### Physiological regulation of SREBP and lipogenesis by feeding and starvation

Previous studies and our new observations suggest a physiological context for the role of Cdk8 in inhibiting SREBP and lipogenesis (Fig. 1A). Insulin signaling and amino acids can activate mTORC1, which can downregulate CDK8 or CycC (Feng et al., 2015; Xie et al., 2015). For simplicity, the following amino acids are numbered based on the equivalent sites in *Drosophila* SREBP. On the one hand, mTORC1 activates p70S6K, which can phosphorylate the SREBP precursors at Ser423, Ser424 and Ser429, thereby protecting SREBP from proteasome degradation and promoting its processing into the nuclear form of SREBP (Dong et al., 2020) (Fig. 1A; Fig. S3C). On the other hand, activated mTORC1 can downregulate CDK8 (Feng et al., 2015; Xie et al., 2015), which allows mature SREBP to stimulate lipogenic gene expression (Zhao et al., 2012) (Fig. 1A). According to this model, under feeding conditions, Cdk8 can no longer inhibit SREBP, thus the gain of either wild-type SREBP or the SREBP^T390A^ mutant can equally stimulate the expression of SREBP target genes (Fig. 4G,H).

Conversely, we have observed that the level of Cdk8 proteins is significantly elevated upon starvation in *Drosophila* larvae, with a concurrent reduction of the nSREBP proteins (Xie et al., 2015). This increase in CDK8 proteins is likely due to the inactivation of mTORC1 (Feng et al., 2015). In addition, it has been reported that starvation or overexpression of TSC1/TSC2 inhibits the activity of mTORC1 and p70S6K (Blagosklonny, 2011; Garami et al., 2003). Low p70S6K activity may lead to hypophosphorylation of Ser423, Ser424 and Ser429 residues of SREBP, thereby reducing the efficiency of the procession of full-length SREBP (Dong et al., 2020) (Fig. 1A). On the other hand, increased CDK8 can phosphorylate SREBP at Thr390 (Zhao et al., 2012), which may be subsequently phosphorylated by GSK-3β at Ser394 (Punga et al., 2006), thereby promoting SREBP degradation (Punga et al., 2006; Zhao et al., 2012) (Fig. 1A). Thus, overexpressed wild-type SREBP can be quickly eliminated during starvation, resulting in unchanged *FAS-EGFP* expression (Fig. 4I). However, the effect of starvation on SREBP activity is abolished by mutating the Thr390 residue, as the phosphodeficient form of SREBP can still activate *FAS-EGFP* transcription under starvation conditions (Fig. 4J). These observations suggest that Cdk8 plays a critical role in inhibiting SREBP-dependent lipogenesis during starvation, and this mechanism is repressed by downregulating Cdk8 protein through mTORC1 under feeding conditions (Fig. 1A). Given that both Cdk8 and GSK-3β are shown to phosphorylate Thr390 of SREBP, it is necessary to further investigate how these different kinases coordinate with each other in phosphorylating SREBP and lipogenesis in different biological contexts *in vivo*.

### Physical interactions between SREBP and Cdk8 or MED15

Our biochemical and yeast two-hybrid analyses suggest that either Cdk8 or MED15 can directly interact with a small fragment within the N-terminus of *Drosophila* SREBP. Mutation of six amino acids (DMLDII, AA36-41) in *Drosophila* SREBP abolished its interaction with either Cdk8 or MED15, suggesting that these six amino acids are essential for mediating these physical interactions. These results further extend the previous report that SREBP in *C. elegans* can interact with MED15 in both *C. elegans* and mammalian cells, and that MED15 is required for SREBP-activated gene expression (Yang et al., 2006).

It is unclear how Cdk8 interacts with the N-terminus of SREBP in *Drosophila*, yet the phosphorylation site (Thr390) is close to the C-terminus of mature SREBP. Except the bHLH DNA-binding domain, most regions of nSREBP, including both the N-terminus and C-terminus, seem to be intrinsically disordered (Fig. 6A). We speculate that the interaction between Cdk8 and the N-terminus of SREBP causes conformational change and brings the kinase domain of Cdk8 close to the C-terminus of SREBP, thereby enabling Cdk8 to phosphorylate the Thr390 residue. Despite the biochemical and *in silico* analyses presented in this study and previous works (Yang et al., 2006; Zhao et al., 2012), evidence showing direct interactions among these proteins *in vivo* is still lacking. Perhaps, solving the structure of the SREBP-CDK8 complex using cryogenic electron microscopy, or three-dimensional structural and simulation analyses, may provide structural insights into this process.

**Fig. 7.**
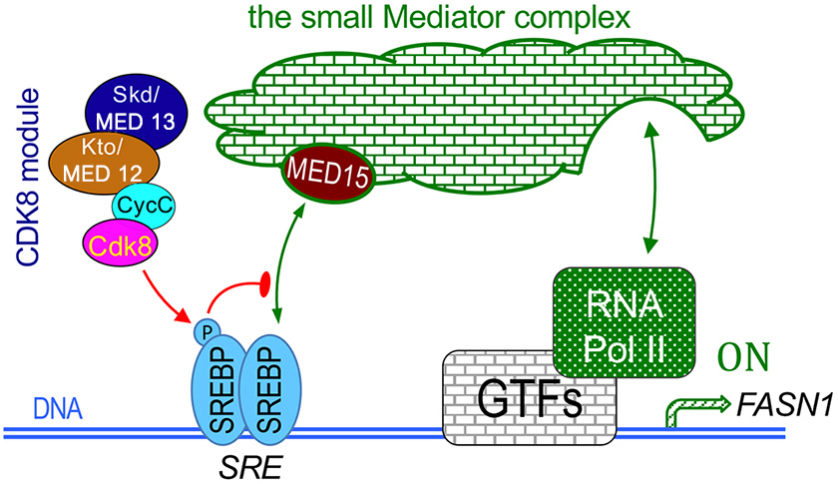
**Working model.** Model of SREBP-dependent transcription through the Cdk8 kinase module (CKM) and the small Mediator complex. The N-terminus of *Drosophila* SREBP (AA25-50) may interact with the small Mediator complex through MED15 subunits, thereby activating the expression of SREBP target genes, such as *FASN1*. Cdk8 may compete with MED15 in binding with the N-terminus of SREBP. Direct interactions between the N-terminus of SREBP (AA25-50) and Cdk8 may enable Cdk8 to phosphorylate the Thr390 residue near the C-terminus of the mature SREBP, thereby turning off SREBP-induced gene expression. See Discussion for details. The *FASN1* gene encodes fatty acid synthase 1. GTFs, general transcription factors; RNA Pol II, RNA polymerase II; SRE, sterol regulatory element.

There are several possible scenarios to explain the dynamic interplay among SREBP, Cdk8 and MED15. Given the current understanding of the role of Mediator complexes and how the CKM regulates the transcription of a few transactivators, the most parsimonious model is that the mature SREBP homodimer binds to the sterol regulatory elements (SREs) of its target genes, and the N-terminus of SREBP directly interacts with the MED15 subunit of the small Mediator complex, which interacts with RNA polymerase II and other general transcription factors, allowing for transcription initiation (Fig. 7). Prior studies suggest that the CKM is recruited to the transcription start sites shortly after the transcription initiation, thereby limiting the subsequent rounds of transcription re-initiation (Fryer et al., 2004; Hahn, 2004). Exactly how the CKM is subsequently recruited to the promoter remains unclear. Interestingly, recent analyses of the budding yeast CKM structure using cryo-electron microscopy have revealed a PIWI domain in MED13 (Li et al., 2021). PIWI proteins bind to RNAs and are known for their roles in regulating RNA interference and transposon silencing (Ross et al., 2014). Thus, we speculate that nascent mRNAs interact with MED13 via its PIWI domain, which facilitates CKM recruitment to the promoter region, where the CKM binds to the small Mediator complex, while CDK8 interacts with specific transactivators such as SREBP. This could explain how the CKM is recruited to promoters after transcription elongation, thereby limiting transcription re-initiation. It is also unclear whether CDK8 directly competes with MED15 in binding to the same N-terminus of SREBP or to two different N-termini of the SREBP homodimer. Subsequently, CDK8 phosphorylates the Thr402 of human SREBP-1C or Thr390 of *Drosophila* SREBP, thereby reducing SREBP binding to the SREs, favoring the export of phospho-SREBPs to the cytoplasm for degradation. In this model, MED15 is necessary for SREBP-activated expression of lipogenic genes, whereas Cdk8 is required for turning this process off (Fig. 7). This model can explain the biological consequences that we observed in *Cdk8* and *CycC* mutants, and our analyses of SREBP^T390A^ mutant proteins provide further support for the dynamic model described above.

A potential caveat of this study is that the experimental evidence for the direct phosphorylation of *Drosophila* SREBP on Thr390 by Cdk8 is lacking; this assumption is inferred based on an *in vitro* CDK8 kinase assay using human SREBP-1C as the substrate and the conservation of the threonine residue in difference species (Zhao et al., 2012). Owing to the lack of phosphospecific antibodies for the *Drosophila* SREBP Thr390 residue, it is also unknown whether SREBP is phosphorylated at the Thr390 residue *in vivo*, and whether manipulation of Cdk8 activity *in vivo* alters the phosphorylation status of this site. Therefore, we still cannot establish beyond doubt that SREBP is a bona fide substrate of Cdk8. However, the functional analyses of the phosphodeficient SREBP summarized in this work further improve confidence for the inhibitory effects of Cdk8 on SREBP and *de novo* lipogenesis, which we proposed previously based on genetic and biochemical analyses in *Drosophila* and mammalian systems (Zhao et al., 2012).

Taken together, this work advances our understanding of how mature SREBP activates its target gene transcription and how this process is turned off. It serves as yet another example of how transcription activation is tightly coupled with its inactivation and explains why transactivators generally have a short half-life (Tansey, 2001). Perhaps, further work using fluorescent-tagged subunits of the Mediator complex and live imaging will help to define the concerted and dynamic actions of the small Mediator complex and the CKM in the tight control of SREBP activities in the nucleus and SREBP-dependent lipogenesis.

## MATERIALS AND METHODS

### Fly maintenance and generation of *UAS-SREBP* transgenic lines

Flies were raised at 25°C on a standard cornmeal, molasses and yeast medium. To construct the *UAS-SREBP* transgenic lines, we first amplified the DNA sequence of the mature SREBP using high-fidelity PrimeSTAR Max DNA polymerase (Takara, R045A) and primers SREBP-5.10 and SREBP-3.10 (Table S1). After purification, the PCR fragment was inserted into the pENTR/D-TOPO vector (Thermo Fisher Scientific, K240020), and then amplified in *Escherichia coli* strain DH5α. The T390A mutation was generated with a QuikChange II Site-Directed Mutagenesis Kit (Agilent Technologies, 200523) with primers SREBP-TA-5.1 and SREBP-TA-3.1 (Table S1). After validation by sequencing, the two pENTR constructs were recombined into the *pVALIUM10-roe* vector (https://fgr.hms.harvard.edu/trip-plasmid-vector-sets), which contains the *attB* sequence for site-specific insertion into the *Drosophila* genome (Perkins et al., 2015) using Gateway LR Clonase II Enzyme mix (Thermo Fisher Scientific, 11791100), following the manufacturer's instructions. The amplified constructs were micro-injected into the integrase-expressing fly strain with *attP2* site (BL-25710: *P{y[+t7.7]=nos-φC31^NLS^}X, y^1^ sc^1^ v^1^ sev^21^; P{y[+t7.7]=CaryP}attP2*) using the service provided by Rainbow Transgenic Flies (Camarillo, CA, USA). To achieve potent expression of genes of interest, the original *pVALIUM10-roe* vector contains ten Gal4-binding sites (10xUAS) with a 5xUAS sequence flanked by two loxP sites (Perkins et al., 2015). We observed that transgenic lines using 10xUAS vector to ectopically express SREBP were too strong and caused early larval lethality when driven with salivary-specific Gal4 lines. Thus, we removed this 5xUAS cassette using the bacteriophage Cre recombinase (NEB, M0298S) *in vitro* and generated *5xUAS-SREBP*^WT^ and *UAS-SREBP^T390A^* transgenic lines with these constructs inserted at the *attP2* landing site. To facilitate detection of these SREBP proteins, we added a V5 tag to the N-terminus of these proteins; primer sequences are included in Table S1.

### Generation of somatic clones in larval fat body and intestines

The first-instar larvae were heat shocked at 37°C for 2 min, and then raised at 25°C until the late-third-instar wandering stage. The fat body was dissected in phosphate-buffered saline (PBS) and fixed in 4% formaldehyde at room temperature for 10 min. For FAS-EGFP imaging, the fat body was incubated with 1 μM 4′,6-diamidino-2-phenylindole (DAPI) in PBS at room temperature for 10 min, washed three times with PBS, and then mounted in VECTASHIELD antifade mounting medium (Vector Laboratories, H-1000). For Nile Red staining, the fat body was washed three times with PBS-Tween 20 for 10 min each, and then incubated with 100 ng/ml Nile Red at room temperature for 30 min, followed by DAPI staining and mounting as described above for FAS-EGFP. We used ImageJ to quantify the levels of FAS-EGFP, and *P*-values were calculated using one-tailed unpaired *t*-tests.

To generate clones in intestines, we first starved 3-day-old adult flies of specific genotypes (as described in the Results section) by keeping them in vials containing 1.5% agar for 24 h, and then performed heat-shock treatment at 37°C for 40 min. These animals were then kept in agar vials for a further 6 h before dissection at 25°C. The control group was kept in vials with normal food but heat shocked and dissected at the same time point. Intestines of these animals were dissected, fixed, stained with DAPI and mounted as described above. Confocal images were taken using a Nikon Ti Eclipse or a Zeiss LSM900 confocal microscope system and then processed by Adobe Photoshop 2021 software.

### Immunostaining and BODIPY staining

Salivary glands from third-instar larvae at the wandering stage were dissected in PBS and fixed in 4% formaldehyde for 10 min at room temperature. After rinsing, the salivary glands were then incubated with BODIPY 493/503 (1 µg/ml, Invitrogen, D3922; diluted in PBS) for 2 h at room temperature. After rising three times in PBS (10 min each), the tissues were mounted in VECTASHIELD medium.

For immunostaining, we used an anti-SREBP antiserum (Xie et al., 2015). Anti-SREBP antibody was purified using Dynabeads Protein A (Thermo Fisher Scientific, 10001D). The fixed fat body was permeabilized with PBS-0.1% Triton X-100 three times, 10 min each, and blocked with PBS+0.1% Triton X-100+5% normal goat serum+0.2% bovine serum albumin (PBS-Triton X-100-NGS-BSA) at room temperature for 1 h. The fat body was then incubated with anti-SREBP (1:100 diluted in PBS-Triton X-100-NGS-BSA) at 4°C overnight. After rinsing with PBS-Triton X-100 three times, the tissues were incubated with a secondary antibody (Alexa Fluor 488 AffiniPure donkey anti-rabbit, Jackson Immunological Laboratories, 711-545-152; 1:1000 diluted in PBS-Triton X-100-NGS-BSA) at room temperature for 1 h, followed by the same DAPI staining, rinsing and mounting steps as described above. Anti-V5 (Invitrogen, R960-25; 1:500) was used for V5-tagged SREBP staining, with secondary antibody (Alexa Fluor 488 AffiniPure goat anti-mouse, Jackson Immunological Laboratories, 115-545-003; 1:1000). ImageJ was used for quantifying the fluorescent intensity of single-plane confocal images.

### RNA-seq sample preparation and data analysis

The total RNA from ten third-instar larvae of *w^1118^* (as the wild-type control) and *Cdk8^K185^* homozygotes at the wandering stage (triplicate for each genotype) was extracted using 1.0 ml TRIzol Regent (Invitrogen), and then purified using an RNeasy Mini Kit (Qiagen, 74104) following the manufacturer's instructions. The preparation and sequencing of the RNA libraries, as well as quality control and adapter trimming, were performed by the Texas A&M University (TAMU) Genomics and Bioinformatics Service using an Illumina HiSeq 2500 following the standard protocols. The processed files were uploaded to and analyzed on the TAMU High Performance Research Computing cluster Terra. RNA-seq sequences were aligned to the *Drosophila melanogaster* genome FlyBase release 6.30 using STAR open source software (Dobin et al., 2013). The gene counts were generated by the featureCounts function from the Subread package (Liao et al., 2014). Differential expression analysis was performed using the DESeq2 R package (Love et al., 2014), with a Benjamini–Hochberg method adjusted *P*-value of 0.05 used to identify statistically significant differentially expressed genes. The heatmaps were generated by the pheatmap R package (https://cran.r-project.org/web/packages/pheatmap/pheatmap.pdf), with normalized gene counts provided by DESeq2. The clusterProfiler R package was used to perform Gene Ontology analysis of significantly altered genes associated with FlyBase gene ID (Yu et al., 2012). The Benjamini–Hochberg multiple testing correction method was used, and the output dot plot was generated by the enrichplot R package (https://bioconductor.org/packages/release/bioc/html/enrichplot.html).

### HCR RNA-FISH

Five pairs of salivary glands from the third-instar larvae were dissected in PBS and then fixed in 4% formaldehyde for 20 min at room temperature. Fixed salivary glands were then rinsed three times in 1× PBS with 0.1% Tween 20 (PBST), 5 min each. After rehydrating the salivary gland in 100%/75%/50%/25% methanol in PBST, the samples were washed three times with PBST and then incubated in 10 μg/ml proteinase K for 5 min. Following three washes in PBST, the samples were fixed again in 4% formaldehyde for 20 min. For the rest of the steps, we followed the multiplexed HCR RNA-FISH protocol provided by Molecular Instruments (https://files.molecularinstruments.com/MI-Protocol-RNAFISH-GenericSolution-Rev7.pdf) with the following modifications: we added DAPI for the first 30 min of 5× sodium chloride sodium citrate with 0.1% Tween 20 washing, then mounted the samples in Antifade Mounting Medium (VECTASHIELD) for microscopy. The probe set for *FANS1* (lot number PRQ415) was used with B1-Alexa Fluor 488 amplifiers, the probe set for *AcCoAS* (lot number PRQ416) was used with B2-Alexa Fluor 594 amplifiers, and the probe set for *ACC* (lot number PRQ417) was used with B3-Alexa Fluor 647 amplifiers (all purchased from Molecular Instruments) in multiplexed *in situ* HCR. Confocal images were taken using a Zeiss LSM900 confocal microscope system and then processed by Adobe Photoshop CS6 software.

### Protein expression and GST pull-down assay

Cdk8 (AA1-262), MED15 (full length) and SREBP fragments were amplified with PrimeSTAR Max premix (Takara, R045A), using the primers listed in Table S1. These fragments were inserted into pENTR/D-TOPO vector (Thermo Fisher Scientific, K240020). pENTR-SREBP-1-A-3 was mutated by a QuikChange II Site-Directed Mutagenesis Kit (Agilent, 200523) using primers SREBP-mut-5.1 and SREBP-mut-3.1 (Table S1). The Cdk8 and MED15 fragments in pENTR vectors were recombined into the pDEST17 vector (N-terminal 6XHis tag) with Gateway LR Clonase II Enzyme mix (Thermo Fisher Scientific, 11791020). Similarly, the SREBP fragments, including mutated pENTR-SREBP-1-A-3, were recombined into the pDEST15 vector (N-terminal GST tag). The constructs were transformed into *E. coli* strain Rosetta for protein expression using standard protocols. Purification of GST-tagged proteins and the GST pull-down assays were performed using the same protocol as described previously (Li et al., 2020).

### Yeast two-hybrid assay

The yeast two-hybrid assay was performed as described before using AH109 yeast strain (Li et al., 2020). pENTR-SREBP-1-A-2 (SREBP1-50) was recombined into pGBKT7-GW vector with Gateway LR Clonase II Enzyme mix, and pENTR-Med15 (full length) was recombined with the pGADT7-GW vector. The pGADT7-CDK8 plasmid was described previously (Li et al., 2020). The SD/−Leu/−Trp/−His medium (MP Biomedicals) was supplemented with 2.5, 5 or 10 mM 3-amino-1,2,4-triazole (3-AT; Sigma-Aldrich, A8056).

### Sequence alignment and analyses of the intrinsically disordered regions of SRBEP proteins

The amino acid sequences of SREBP proteins from different species were downloaded from the National Center for Biotechnology Information (NCBI) Reference Sequence (RefSeq) database and aligned using Clustal Omega with default parameters (https://www.ebi.ac.uk/Tools/msa/clustalo/) (O'Leary et al., 2016; Sievers et al., 2011). The SREBP sequences of different species in FASTA format were individually uploaded to https://iupred2a.elte.hu/ and performed ANCHOR2 and IUPred2A predictions with default parameters (Dosztanyi et al., 2009; Mészáros et al., 2018). The heatmaps were drawn by pheatmap R package (https://cran.r-project.org/web/packages/pheatmap/pheatmap.pdf), based on the predicted scores.

### Statistical analyses

*P*-values were calculated using one-tailed Type 3 *t*-tests, and error bars indicate s.d. The numbers of samples analyzed are indicated in Fig. 2H and Fig. 3E.

## Supplementary Material

10.1242/dmm.049650_sup1 Supplementary information

## References

[DMM049650C1] Allen, B. L. and Taatjes, D. J. (2015). The Mediator complex: a central integrator of transcription. *Nat. Rev. Mol. Cell Biol.* 16, 155-166. 10.1038/nrm3951 25693131 PMC4963239

[DMM049650C2] Amemiya-Kudo, M., Shimano, H., Hasty, A. H., Yahagi, N., Yoshikawa, T., Matsuzaka, T., Okazaki, H., Tamura, Y., Iizuka, Y., Ohashi, K. et al. (2002). Transcriptional activities of nuclear SREBP-1a, -1c, and -2 to different target promoters of lipogenic and cholesterogenic genes. *J. Lipid Res.* 43, 1220-1235. 10.1194/jlr.M100417-JLR200 12177166

[DMM049650C3] Baenke, F., Peck, B., Miess, H. and Schulze, A. (2013). Hooked on fat: the role of lipid synthesis in cancer metabolism and tumour development. *Dis. Model. Mech.* 6, 1353-1363. 10.1242/dmm.011338 24203995 PMC3820259

[DMM049650C4] Blagosklonny, M. V. (2011). Rapamycin-induced glucose intolerance: hunger or starvation diabetes. *Cell Cycle* 10, 4217-4224. 10.4161/cc.10.24.18595 22157190

[DMM049650C5] Brown, M. S. and Goldstein, J. L. (1997). The SREBP pathway: regulation of cholesterol metabolism by proteolysis of a membrane-bound transcription factor. *Cell* 89, 331-340. 10.1016/S0092-8674(00)80213-5 9150132

[DMM049650C6] Carvalho, M., Schwudke, D., Sampaio, J. L., Palm, W., Riezman, I., Dey, G., Gupta, G. D., Mayor, S., Riezman, H., Shevchenko, A. et al. (2010). Survival strategies of a sterol auxotroph. *Development* 137, 3675-3685. 10.1242/dev.044560 20940226 PMC2964098

[DMM049650C7] Cheng, C., Geng, F., Cheng, X. and Guo, D. (2018). Lipid metabolism reprogramming and its potential targets in cancer. *Cancer Commun. (Lond)* 38, 27. 10.1186/s40880-018-0301-4 29784041 PMC5993136

[DMM049650C8] Choi, H. M., Chang, J. Y., Trinh, L. A., Padilla, J. E., Fraser, S. E. and Pierce, N. A. (2010). Programmable in situ amplification for multiplexed imaging of mRNA expression. *Nat. Biotechnol.* 28, 1208-1212. 10.1038/nbt.1692 21037591 PMC3058322

[DMM049650C9] Choi, H. M. T., Schwarzkopf, M., Fornace, M. E., Acharya, A., Artavanis, G., Stegmaier, J., Cunha, A. and Pierce, N. A. (2018). Third-generation in situ hybridization chain reaction: multiplexed, quantitative, sensitive, versatile, robust. *Development* 145, dev165753. 10.1242/dev.165753 29945988 PMC6031405

[DMM049650C10] Dannappel, M. V., Sooraj, D., Loh, J. J. and Firestein, R. (2018). Molecular and in vivo functions of the CDK8 and CDK19 kinase modules. *Front. Cell Dev. Biol.* 6, 171. 10.3389/fcell.2018.00171 30693281 PMC6340071

[DMM049650C11] Desvergne, B., Michalik, L. and Wahli, W. (2006). Transcriptional regulation of metabolism. *Physiol. Rev.* 86, 465-514. 10.1152/physrev.00025.2005 16601267

[DMM049650C12] Dirks, R. M. and Pierce, N. A. (2004). Triggered amplification by hybridization chain reaction. *Proc. Natl. Acad. Sci. USA* 101, 15275-15278. 10.1073/pnas.0407024101 15492210 PMC524468

[DMM049650C13] Dobin, A., Davis, C. A., Schlesinger, F., Drenkow, J., Zaleski, C., Jha, S., Batut, P., Chaisson, M. and Gingeras, T. R. (2013). STAR: ultrafast universal RNA-seq aligner. *Bioinformatics* 29, 15-21. 10.1093/bioinformatics/bts635 23104886 PMC3530905

[DMM049650C14] Dobrosotskaya, I. Y., Seegmiller, A. C., Brown, M. S., Goldstein, J. L. and Rawson, R. B. (2002). Regulation of SREBP processing and membrane lipid production by phospholipids in Drosophila. *Science* 296, 879-883. 10.1126/science.1071124 11988566

[DMM049650C15] Dong, Q., Giorgianni, F., Deng, X., Beranova-Giorgianni, S., Bridges, D., Park, E. A., Raghow, R. and Elam, M. B. (2014). Phosphorylation of sterol regulatory element binding protein-1a by protein kinase A (PKA) regulates transcriptional activity. *Biochem. Biophys. Res. Commun.* 449, 449-454. 10.1016/j.bbrc.2014.05.046 24853806 PMC4670028

[DMM049650C16] Dong, Q., Giorgianni, F., Beranova-Giorgianni, S., Deng, X., O'Meally, R. N., Bridges, D., Park, E. A., Cole, R. N., Elam, M. B. and Raghow, R. (2015). Glycogen synthase kinase-3-mediated phosphorylation of serine 73 targets sterol response element binding protein-1c (SREBP-1c) for proteasomal degradation. *Biosci. Rep.* 36, e00284. 10.1042/BSR20150234 26589965 PMC4718510

[DMM049650C17] Dong, Q., Majumdar, G., O'Meally, R. N., Cole, R. N., Elam, M. B. and Raghow, R. (2020). Insulin-induced de novo lipid synthesis occurs mainly via mTOR-dependent regulation of proteostasis of SREBP-1c. *Mol. Cell. Biochem.* 463, 13-31. 10.1007/s11010-019-03625-5 31541353

[DMM049650C18] Dosztanyi, Z., Meszaros, B. and Simon, I. (2009). ANCHOR: web server for predicting protein binding regions in disordered proteins. *Bioinformatics* 25, 2745-2746. 10.1093/bioinformatics/btp518 19717576 PMC2759549

[DMM049650C19] Edgar, B. A. (2006). How flies get their size: genetics meets physiology. *Nat. Rev. Genet.* 7, 907-916. 10.1038/nrg1989 17139322

[DMM049650C76] Edgar, R., Domrachev, M. and Lash, A. E. (2002). Gene Expression Omnibus: NCBI gene expression and hybridization array data repository. *Nucleic Acids Res*. 30, 207-210. 10.1093/nar/30.1.207 11752295 PMC99122

[DMM049650C20] Fahy, E., Subramaniam, S., Murphy, R. C., Nishijima, M., Raetz, C. R. H., Shimizu, T., Spener, F., van Meer, G., Wakelam, M. J. O. and Dennis, E. A. (2009). Update of the LIPID MAPS comprehensive classification system for lipids. *J. Lipid Res.* 50 Suppl., S9-S14. 10.1194/jlr.R800095-JLR200 19098281 PMC2674711

[DMM049650C21] Feng, D., Youn, D. Y., Zhao, X., Gao, Y., Quinn, W. J., III, Xiaoli, A. M., Sun, Y., Birnbaum, M. J., Pessin, J. E. and Yang, F. (2015). mTORC1 down-regulates Cyclin-Dependent Kinase 8 (CDK8) and Cyclin C (CycC). *PLoS ONE* 10, e0126240. 10.1371/journal.pone.0126240 26042770 PMC4456374

[DMM049650C22] Fryer, C. J., White, J. B. and Jones, K. A. (2004). Mastermind recruits CycC:CDK8 to phosphorylate the Notch ICD and coordinate activation with turnover. *Mol. Cell* 16, 509-520. 10.1016/j.molcel.2004.10.014 15546612

[DMM049650C23] Gao, X., Xie, X.-J., Hsu, F.-N., Li, X., Liu, M., Hemba-Waduge, R.-U., Xu, W. and Ji, J.-Y. (2018). CDK8 mediates the dietary effects on developmental transition in Drosophila. *Dev. Biol.* 444, 62-70. 10.1016/j.ydbio.2018.10.001 30352217 PMC6263851

[DMM049650C24] Garami, A., Zwartkruis, F. J. T., Nobukuni, T., Joaquin, M., Roccio, M., Stocker, H., Kozma, S. C., Hafen, E., Bos, J. L. and Thomas, G. (2003). Insulin activation of Rheb, a mediator of mTOR/S6K/4E-BP signaling, is inhibited by TSC1 and 2. *Mol. Cell* 11, 1457-1466. 10.1016/S1097-2765(03)00220-X 12820960

[DMM049650C25] Germani, F., Bergantinos, C. and Johnston, L. A. (2018). Mosaic analysis in Drosophila. *Genetics* 208, 473-490. 10.1534/genetics.117.300256 29378809 PMC5788516

[DMM049650C26] Goldstein, J. L., Rawson, R. B. and Brown, M. S. (2002). Mutant mammalian cells as tools to delineate the sterol regulatory element-binding protein pathway for feedback regulation of lipid synthesis. *Arch. Biochem. Biophys.* 397, 139-148. 10.1006/abbi.2001.2615 11795864

[DMM049650C27] Guo, D., Bell, E. H., Mischel, P. and Chakravarti, A. (2014). Targeting SREBP-1-driven lipid metabolism to treat cancer. *Curr. Pharm. Des.* 20, 2619-2626. 10.2174/13816128113199990486 23859617 PMC4148912

[DMM049650C28] Hahn, S. (2004). Structure and mechanism of the RNA polymerase II transcription machinery. *Nat. Struct. Mol. Biol.* 11, 394-403. 10.1038/nsmb763 15114340 PMC1189732

[DMM049650C29] Hannah, V. C., Ou, J., Luong, A., Goldstein, J. L. and Brown, M. S. (2001). Unsaturated fatty acids down-regulate srebp isoforms 1a and 1c by two mechanisms in HEK-293 cells. *J. Biol. Chem.* 276, 4365-4372. 10.1074/jbc.M007273200 11085986

[DMM049650C30] Jaluria, P., Konstantopoulos, K., Betenbaugh, M. and Shiloach, J. (2007). A perspective on microarrays: current applications, pitfalls, and potential uses. *Microb. Cell Fact.* 6, 4. 10.1186/1475-2859-6-4 17254338 PMC1796898

[DMM049650C31] Jeon, T.-I. and Osborne, T. F. (2012). SREBPs: metabolic integrators in physiology and metabolism. *Trends Endocrinol. Metab.* 23, 65-72. 10.1016/j.tem.2011.10.004 22154484 PMC3273665

[DMM049650C32] Jewell, J. L., Russell, R. C. and Guan, K.-L. (2013). Amino acid signalling upstream of mTOR. *Nat. Rev. Mol. Cell Biol.* 14, 133-139. 10.1038/nrm3522 23361334 PMC3988467

[DMM049650C33] Knebel, B., Lehr, S., Hartwig, S., Haas, J., Kaber, G., Dicken, H.-D., Susanto, F., Bohne, L., Jacob, S., Nitzgen, U. et al. (2014). Phosphorylation of sterol regulatory element-binding protein (SREBP)-1c by p38 kinases, ERK and JNK influences lipid metabolism and the secretome of human liver cell line HepG2. *Arch. Physiol. Biochem.* 120, 216-227. 10.3109/13813455.2014.973418 25353341

[DMM049650C34] Kotzka, J., Knebel, B., Haas, J., Kremer, L., Jacob, S., Hartwig, S., Nitzgen, U. and Muller-Wieland, D. (2012). Preventing phosphorylation of sterol regulatory element-binding protein 1a by MAP-kinases protects mice from fatty liver and visceral obesity. *PLoS ONE* 7, e32609. 10.1371/journal.pone.0032609 22384276 PMC3287979

[DMM049650C35] Li, Y., Xu, S., Mihaylova, M. M., Zheng, B., Hou, X., Jiang, B., Park, O., Luo, Z., Lefai, E., Shyy, J. Y.-J. et al. (2011). AMPK phosphorylates and inhibits SREBP activity to attenuate hepatic steatosis and atherosclerosis in diet-induced insulin-resistant mice. *Cell Metab.* 13, 376-388. 10.1016/j.cmet.2011.03.009 21459323 PMC3086578

[DMM049650C36] Li, X., Liu, M. and Ji, J.-Y. (2019). Understanding obesity as a risk factor for uterine tumors using Drosophila. *Adv. Exp. Med. Biol.* 1167, 129-155. 10.1007/978-3-030-23629-8_8 31520353

[DMM049650C37] Li, X., Liu, M., Ren, X., Loncle, N., Wang, Q., Hemba-Waduge, R.-U., Yu, S. H., Boube, M., Bourbon, H.-M. G., Ni, J.-Q. et al. (2020). The mediator CDK8-cyclin C complex modulates Dpp signaling in Drosophila by stimulating Mad-dependent transcription. *PLoS Genet.* 16, e1008832. 10.1371/journal.pgen.1008832 32463833 PMC7282676

[DMM049650C38] Li, Y.-C., Chao, T.-C., Kim, H. J., Cholko, T., Chen, S.-F., Li, G., Snyder, L., Nakanishi, K., Chang, C.-E., Murakami, K. et al. (2021). Structure and noncanonical Cdk8 activation mechanism within an Argonaute-containing Mediator kinase module. *Sci. Adv.* 7, eabd4484. 10.1126/sciadv.abd4484 33523904 PMC7810384

[DMM049650C39] Liao, Y., Smyth, G. K. and Shi, W. (2014). featureCounts: an efficient general purpose program for assigning sequence reads to genomic features. *Bioinformatics* 30, 923-930. 10.1093/bioinformatics/btt656 24227677

[DMM049650C40] Love, M. I., Huber, W. and Anders, S. (2014). Moderated estimation of fold change and dispersion for RNA-seq data with DESeq2. *Genome Biol.* 15, 550. 10.1186/s13059-014-0550-8 25516281 PMC4302049

[DMM049650C41] Lu, M. and Shyy, J. Y.-J. (2006). Sterol regulatory element-binding protein 1 is negatively modulated by PKA phosphorylation. *Am. J. Physiol. Cell Physiol.* 290, C1477-C1486. 10.1152/ajpcell.00374.2005 16381800

[DMM049650C42] Luo, W. and Brouwer, C. (2013). Pathview: an R/Bioconductor package for pathway-based data integration and visualization. *Bioinformatics* 29, 1830-1831. 10.1093/bioinformatics/btt285 23740750 PMC3702256

[DMM049650C43] Menendez, J. A. and Lupu, R. (2007). Fatty acid synthase and the lipogenic phenotype in cancer pathogenesis. *Nat. Rev. Cancer* 7, 763-777. 10.1038/nrc2222 17882277

[DMM049650C44] Mészáros, B., Erdős, G. and Dosztányi, Z. (2018). IUPred2A: context-dependent prediction of protein disorder as a function of redox state and protein binding. *Nucleic Acids Res.* 46, W329-W337. 10.1093/nar/gky384 29860432 PMC6030935

[DMM049650C45] Moslehi, A. and Hamidi-Zad, Z. (2018). Role of SREBPs in liver diseases: a mini-review. *J. Clin. Transl. Hepatol.* 6, 332-338. 10.14218/JCTH.2017.00061 30271747 PMC6160306

[DMM049650C46] Ni, J. Q., Liu, L. P., Binari, R., Hardy, R., Shim, H. S., Cavallaro, A., Booker, M., Pfeiffer, B. D., Markstein, M., Wang, H. et al. (2009). A Drosophila resource of transgenic RNAi lines for neurogenetics. *Genetics* 182, 1089-1100. 10.1534/genetics.109.103630 19487563 PMC2728850

[DMM049650C47] O'Leary, N. A., Wright, M. W., Brister, J. R., Ciufo, S., Haddad, D., McVeigh, R., Rajput, B., Robbertse, B., Smith-White, B., Ako-Adjei, D. et al. (2016). Reference sequence (RefSeq) database at NCBI: current status, taxonomic expansion, and functional annotation. *Nucleic Acids Res.* 44, D733-D745. 10.1093/nar/gkv1189 26553804 PMC4702849

[DMM049650C48] Osborne, T. F. and Espenshade, P. J. (2009). Evolutionary conservation and adaptation in the mechanism that regulates SREBP action: what a long, strange tRIP it's been. *Genes Dev.* 23, 2578-2591. 10.1101/gad.1854309 19933148 PMC2779761

[DMM049650C49] Parraga, A., Bellsolell, L., Ferré-D'Amaré, A. R. and Burley, S. K. (1998). Co-crystal structure of sterol regulatory element binding protein 1a at 2.3 Å resolution. *Structure* 6, 661-672. 10.1016/S0969-2126(98)00067-7 9634703

[DMM049650C50] Perkins, L. A., Holderbaum, L., Tao, R., Hu, Y., Sopko, R., McCall, K., Yang-Zhou, D., Flockhart, I., Binari, R., Shim, H.-S. et al. (2015). The transgenic RNAi project at Harvard Medical School: resources and validation. *Genetics* 201, 843-852. 10.1534/genetics.115.180208 26320097 PMC4649654

[DMM049650C51] Punga, T., Bengoechea-Alonso, M. T. and Ericsson, J. (2006). Phosphorylation and ubiquitination of the transcription factor sterol regulatory element-binding protein-1 in response to DNA binding. *J. Biol. Chem.* 281, 25278-25286. 10.1074/jbc.M604983200 16825193

[DMM049650C52] Raghow, R., Dong, Q. and Elam, M. B. (2019). Phosphorylation dependent proteostasis of sterol regulatory element binding proteins. *Biochim. Biophys. Acta Mol. Cell Biol. Lipids* 1864, 1145-1156. 10.1016/j.bbalip.2019.04.015 31067497

[DMM049650C53] Rawson, R. B. (2003). The SREBP pathway--insights from Insigs and insects. *Nat. Rev. Mol. Cell Biol.* 4, 631-640. 10.1038/nrm1174 12923525

[DMM049650C54] Rawson, R. B., DeBose-Boyd, R., Goldstein, J. L. and Brown, M. S. (1999). Failure to cleave sterol regulatory element-binding proteins (SREBPs) causes cholesterol auxotrophy in Chinese hamster ovary cells with genetic absence of SREBP cleavage-activating protein. *J. Biol. Chem.* 274, 28549-28556. 10.1074/jbc.274.40.28549 10497220

[DMM049650C55] Rosenfeld, J. M. and Osborne, T. F. (1998). HLH106, a Drosophila sterol regulatory element-binding protein in a natural cholesterol auxotroph. *J. Biol. Chem.* 273, 16112-16121. 10.1074/jbc.273.26.16112 9632664

[DMM049650C56] Ross, R. J., Weiner, M. M. and Lin, H. (2014). PIWI proteins and PIWI-interacting RNAs in the soma. *Nature* 505, 353-359. 10.1038/nature12987 24429634 PMC4265809

[DMM049650C57] Sabatini, D. M. (2017). Twenty-five years of mTOR: Uncovering the link from nutrients to growth. *Proc. Natl. Acad. Sci. USA* 114, 11818-11825. 10.1073/pnas.1716173114 29078414 PMC5692607

[DMM049650C58] Schneider, E. V., Böttcher, J., Blaesse, M., Neumann, L., Huber, R. and Maskos, K. (2011). The Structure of CDK8/CycC Implicates Specificity in the CDK/Cyclin Family and Reveals Interaction with a Deep Pocket Binder. *J. Mol. Biol.* 412, 251-266. 10.1016/j.jmb.2011.07.020 21806996

[DMM049650C59] Seegmiller, A. C., Dobrosotskaya, I., Goldstein, J. L., Ho, Y. K., Brown, M. S. and Rawson, R. B. (2002). The SREBP pathway in Drosophila: regulation by palmitate, not sterols. *Dev. Cell* 2, 229-238. 10.1016/S1534-5807(01)00119-8 11832248

[DMM049650C60] Shimano, H. and Sato, R. (2017). SREBP-regulated lipid metabolism: convergent physiology — divergent pathophysiology. *Nat. Rev. Endocrinol.* 13, 710-730. 10.1038/nrendo.2017.91 28849786

[DMM049650C61] Shimano, H., Yahagi, N., Amemiya-Kudo, M., Hasty, A. H., Osuga, J., Tamura, Y., Shionoiri, F., Iizuka, Y., Ohashi, K., Harada, K. et al. (1999). Sterol regulatory element-binding protein-1 as a key transcription factor for nutritional induction of lipogenic enzyme genes. *J. Biol. Chem.* 274, 35832-35839. 10.1074/jbc.274.50.35832 10585467

[DMM049650C62] Shimomura, I., Bashmakov, Y., Ikemoto, S., Horton, J. D., Brown, M. S. and Goldstein, J. L. (1999). Insulin selectively increases SREBP-1c mRNA in the livers of rats with streptozotocin-induced diabetes. *Proc. Natl. Acad. Sci. USA* 96, 13656-13661. 10.1073/pnas.96.24.13656 10570128 PMC24120

[DMM049650C63] Sievers, F., Wilm, A., Dineen, D., Gibson, T. J., Karplus, K., Li, W., Lopez, R., McWilliam, H., Remmert, M., Söding, J. et al. (2011). Fast, scalable generation of high-quality protein multiple sequence alignments using Clustal Omega. *Mol. Syst. Biol.* 7, 539. 10.1038/msb.2011.75 21988835 PMC3261699

[DMM049650C64] Soutourina, J. (2018). Transcription regulation by the Mediator complex. *Nat. Rev. Mol. Cell Biol.* 19, 262-274. 10.1038/nrm.2017.115 29209056

[DMM049650C65] Sundqvist, A., Bengoechea-Alonso, M. T., Ye, X., Lukiyanchuk, V., Jin, J., Harper, J. W. and Ericsson, J. (2005). Control of lipid metabolism by phosphorylation-dependent degradation of the SREBP family of transcription factors by SCF(Fbw7). *Cell Metab.* 1, 379-391. 10.1016/j.cmet.2005.04.010 16054087

[DMM049650C66] Tang, H.-W., Hu, Y., Chen, C.-L., Xia, B., Zirin, J., Yuan, M., Asara, J. M., Rabinow, L. and Perrimon, N. (2018). The TORC1-regulated CPA complex rewires an RNA processing network to drive autophagy and metabolic reprogramming. *Cell Metab.* 27, 1040-1054.e8. 10.1016/j.cmet.2018.02.023 29606597 PMC6100782

[DMM049650C67] Tansey, W. P. (2001). Transcriptional activation: risky business. *Genes Dev.* 15, 1045-1050. 10.1101/gad.896501 11331599

[DMM049650C68] Wu, X., Yan, R., Cao, P., Qian, H. and Yan, N. (2022). Structural advances in sterol-sensing domain-containing proteins. *Trends Biochem. Sci.* 47, 289-300. 10.1016/j.tibs.2021.12.005 35012873

[DMM049650C69] Xie, X.-J., Hsu, F.-N., Gao, X., Xu, W., Ni, J.-Q., Xing, Y., Huang, L., Hsiao, H.-C., Zheng, H., Wang, C. et al. (2015). CDK8-Cyclin C mediates nutritional regulation of developmental transitions through the ecdysone receptor in Drosophila. *PLoS Biol.* 13, e1002207. 10.1371/journal.pbio.1002207 26222308 PMC4519132

[DMM049650C70] Xu, W., Amire-Brahimi, B., Xie, X.-J., Huang, L. and Ji, J.-Y. (2014). All-atomic molecular dynamic studies of human CDK8: insight into the A-loop, point mutations and binding with its partner CycC. *Comput. Biol. Chem.* 51, 1-11. 10.1016/j.compbiolchem.2014.03.003 24754906 PMC4122639

[DMM049650C71] Yang, F., Vought, B. W., Satterlee, J. S., Walker, A. K., Jim Sun, Z.-Y., Watts, J. L., DeBeaumont, R., Saito, R. M., Hyberts, S. G., Yang, S. et al. (2006). An ARC/Mediator subunit required for SREBP control of cholesterol and lipid homeostasis. *Nature* 442, 700-704. 10.1038/nature04942 16799563

[DMM049650C72] Yin, J.-W. and Wang, G. (2014). The Mediator complex: a master coordinator of transcription and cell lineage development. *Development* 141, 977-987. 10.1242/dev.098392 24550107

[DMM049650C73] Yu, G., Wang, L.-G., Han, Y. and He, Q.-Y. (2012). clusterProfiler: an R package for comparing biological themes among gene clusters. *OMICS* 16, 284-287. 10.1089/omi.2011.0118 22455463 PMC3339379

[DMM049650C74] Zhao, X. and Yang, F. (2012). Regulation of SREBP-mediated gene expression. *Sheng Wu Wu Li Hsueh Bao* 28, 287-294. 10.3724/SP.J.1260.2012.20034 23730104 PMC3667598

[DMM049650C75] Zhao, X., Feng, D., Wang, Q., Abdulla, A., Xie, X.-J., Zhou, J., Sun, Y., Yang, E. S., Liu, L.-P., Vaitheesvaran, B. et al. (2012). Regulation of lipogenesis by cyclin-dependent kinase 8-mediated control of SREBP-1. *J. Clin. Invest.* 122, 2417-2427. 10.1172/JCI61462 22684109 PMC3386818

