# Supplementary material for: Cdk8 attenuates lipogenesis by inhibiting SREBP-dependent transcription in Drosophila
Source: Dis Model Mech. 2022 Nov 14;15(11):dmm049650. doi: 10.1242/dmm.049650 (PMC9702540; doi:10.1242/dmm.049650)
Supplement: Supplementary information [file dmm-15-049650-s1.pdf]

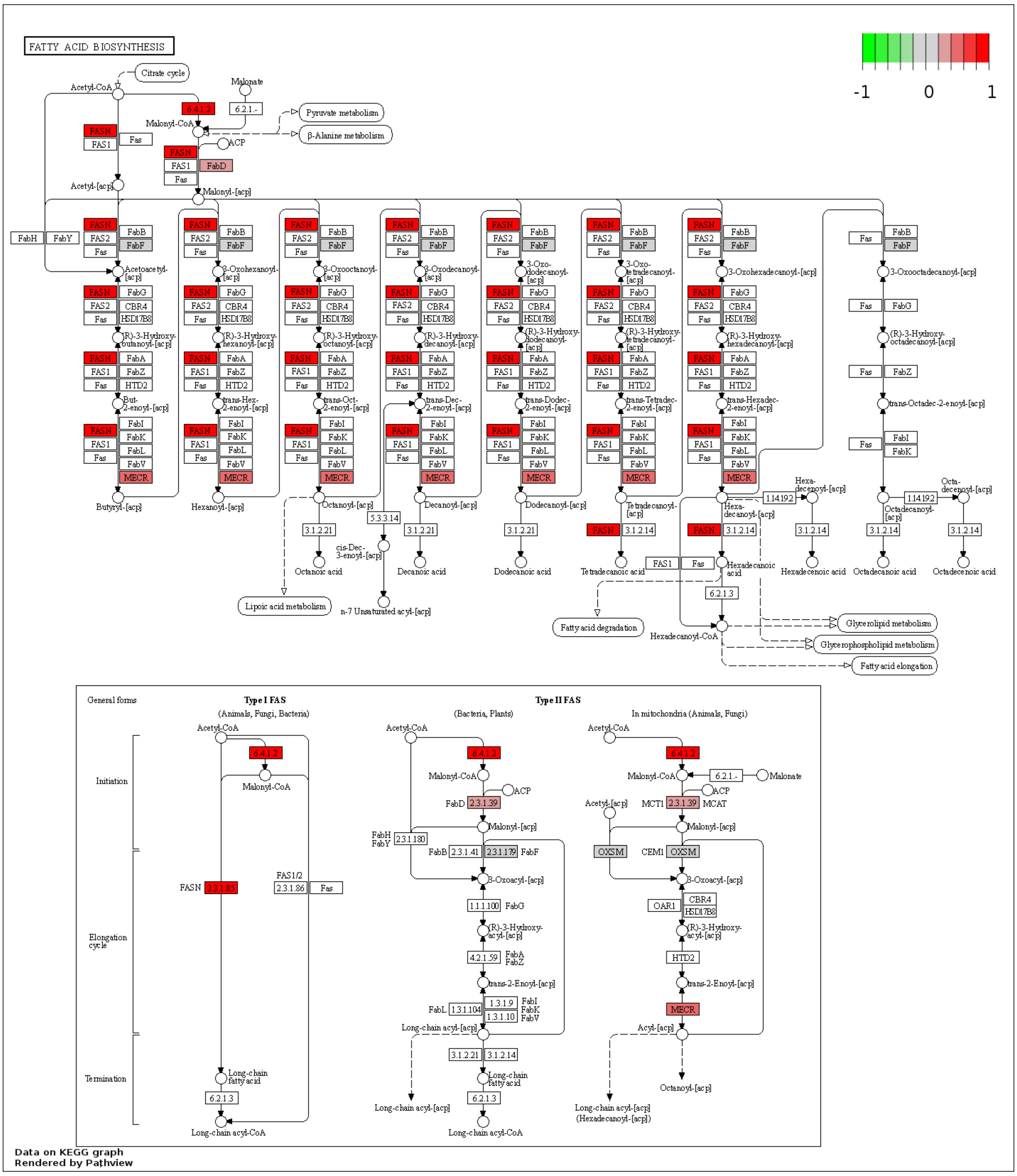

Fig. S1. Mapping of the differentially expressed genes in *cdk*^{K185} mutant larvae *vs.* the control larvae (*w*^{1118}) to the fatty acid synthesis pathway.

Disease Models & Mechanisms • Supplementary information

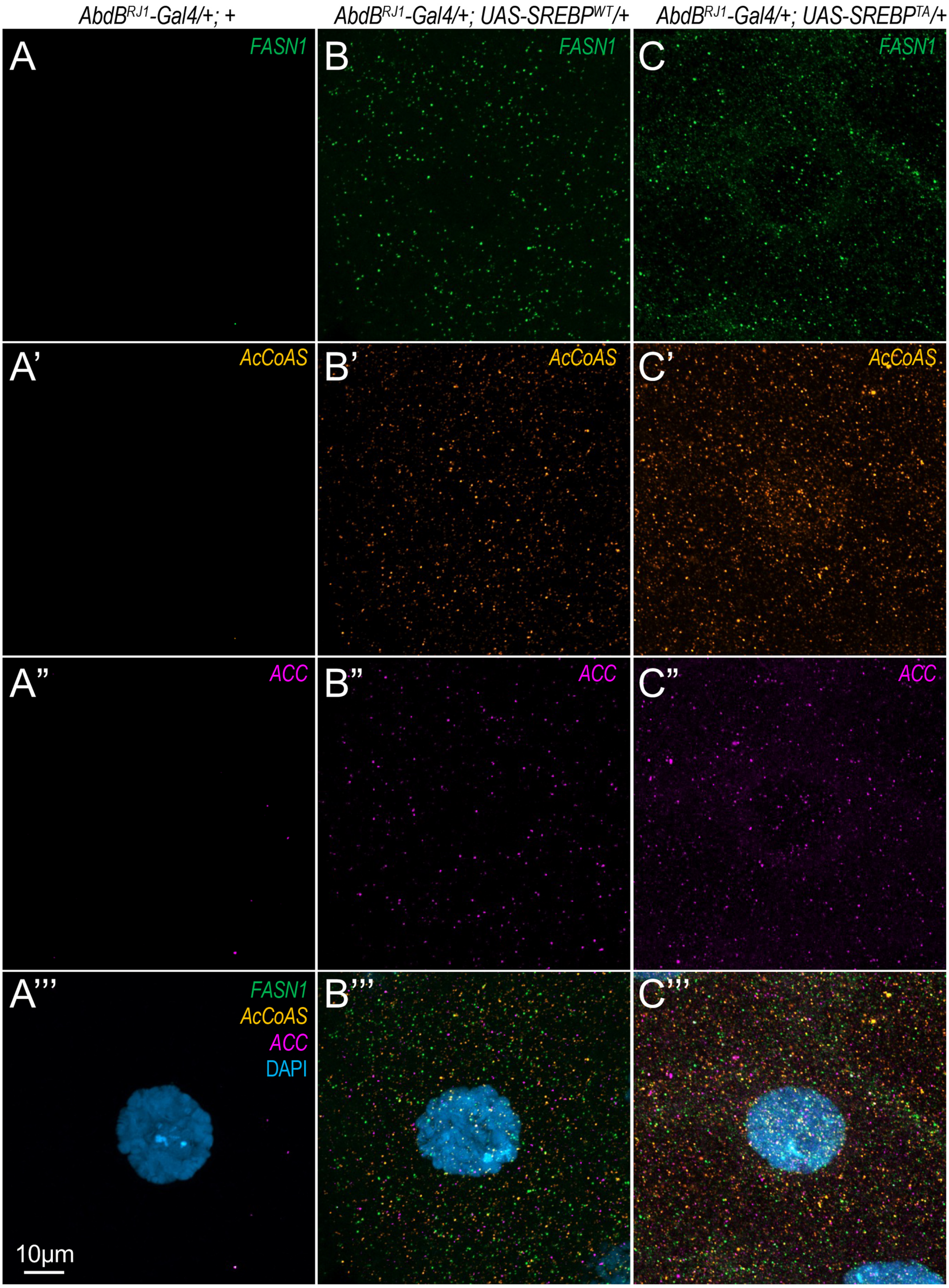

Fig. S2. Detection of SREBP target gene expression in larval salivary glands by multiplexed *in situ* HCR technology. Part of the merged images were shown in Fig. 3F-3H, and individual fluorescent channels for different genes are separately displayed here to help illustrate the difference. Different mRNA transcripts are shown in different colors: *FASN1* (green; B1-Alexa Fluor 488 amplifier), *AcCoAS* (orange; B2-Alexa Fluor 594), and *ACC* (magenta; B3-Alexa Fluor 647). Genotypes: (A) *AbdB^{RJ1}-Gal4/+; +* (control); (B) *AbdB^{RJ1}-Gal4/+; UAS-SREBP^{+}/+*; (C) *AbdB^{RJ1}-Gal4/+; UAS-SREBP^{TA}/+*. Merged images with DAPI (blue) are shown at the bottom row (A'''-C''').

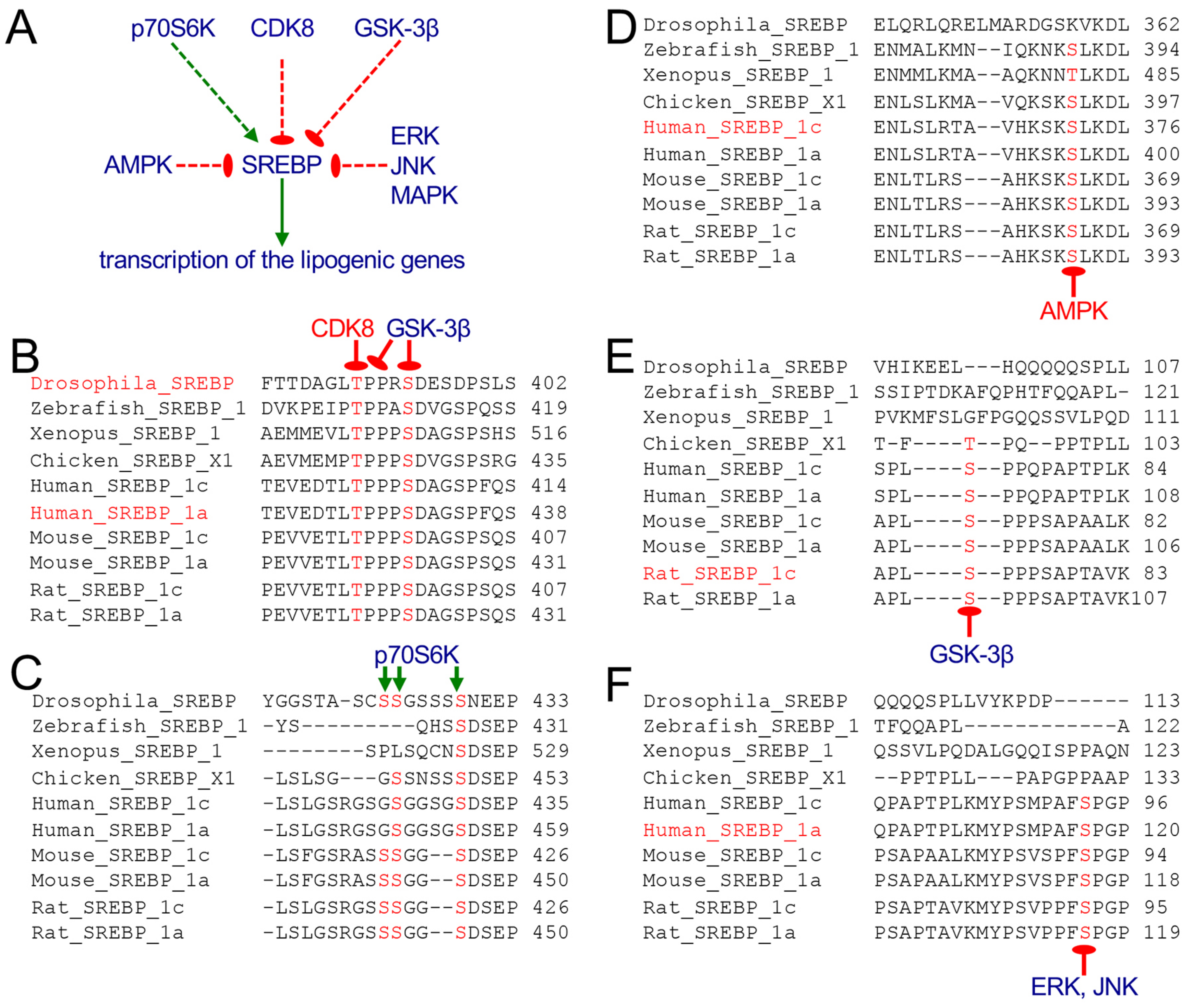

Fig. S3. Aligned sequence of amino acid residues around the potential phosphorylation sites of SREBP from indicated species. (A) Summary of the known kinases that can phosphorylate SREBPs. The phosphorylation sites for CDK8 (B), GSK3β (B), and p70S6K (C) appear to be highly conserved in SREBPs from flies to humans, while the phosphorylation sites for AMPK (D), GSK3β (E), and ERK/JNK (F) are less conserved. Arrow, activation; blunt arrows, inhibition.

Disease Models & Mechanisms • Supplementary information

Table S1. List of primer sequences used in this study

Primer name	Primer sequence (5' to 3')	Notes
dCDK8-N-5.1	CACCATGGACTACGATTTCAAGAT	Fig. 5
dCDK8-N-3.1	CTACGGAAACCCCATCACATTGA	Fig. 5
dSREBP-5.10	CACCATGGACACGACACTGATGAAC	Fig. 5
dSREBP-3.11	CTAGGCCCGACTACCTGTGCTTG	Fig. 5
dSREBP-5.12	CACCATGCAGTCGTATCCGCAACCCTT	Fig. 5
dSREBP-3.12	CTACTCCCTCTGCAGGCGCTG	Fig. 5
dSREBP-5.13	CACCATGGACAAGATTAACGAGTTGAA	Fig. 5
dSREBP-3.10	GAGTCCGAGGCGAGAGTG	Fig. 5
dSREBP-3.21	CTACTGCTGATGCAGTTCCTCCT	Fig. 5
dSREBP-5.22	CACCATGCAGATGTACAACATGCTGCT	Fig. 5
dSREBP-3.22	CTACTGGGGCAAAGGAGCAGAAG	Fig. 5
dSREBP-5.23	CACCATGACGGCGGTGTATCCCCCATC	Fig. 5
dSREBP-3.31	CTAGACATCCTCCGCTTTGAACA	Fig. 5
dSREBP-3.32	CTACGTCGGCGCCAGGTCCATGT	Fig. 5
dSREBP-3.33	CTATTGCGGCTGCTGATCCACGG	Fig. 5
dSREBP-mut-5.1	GCGCCAGGTCCATGTCGTTGAGGGCGGCGGCCGCCGCGGCCGAGTGCAGGTCGGCGTCGAAC	Fig. 6
dSREBP-mut-3.1	GTTCGACGCCGACCTGCACTCGGCCGCGGCGGCCGCCGCCCTCAACGACATGGACCTGGCGC	Fig. 6
dSREBP-TA-5.1	ACGGATGCCGGACTGGCGCCGCCACGCAGCGAT	Fig. 2
dSREBP-TA-3.1	ATCGCTGCGTGGCGGCGCCAGTCCGGCATCCGT	Fig. 2
V5-dSREBP-5.1	CACCGCTAGCATGGGTAAGCCTATCCCTAACCCTCTCCTCGGTCTCGATTCTACGGACACGACAC TGATGAACTT	Fig. 2/3
dSREBP-3.1	ctaGAGTCCGAGGCGAGAGTGG	Fig. 2/3
